# Small RNA Profiling of Susceptible and Resistant *Ty-1* Encoding Tomato Plants Upon Tomato Yellow Leaf Curl Virus Infection

**DOI:** 10.3389/fpls.2021.757165

**Published:** 2021-11-18

**Authors:** Corien M. Voorburg, Yuling Bai, Richard Kormelink

**Affiliations:** ^1^Laboratory of Virology, Department of Plant Sciences, Wageningen University and Research, Wageningen, Netherlands; ^2^Plant Breeding, Department of Plant Sciences, Wageningen University and Research, Wageningen, Netherlands

**Keywords:** resistance, geminivirus, *Ty-1*, PTGS, TGS, RNAi, RDR, TYLCV

## Abstract

*Ty-1* presents an atypical dominant resistance gene that codes for an RNA-dependent RNA polymerase (RDR) of the gamma class and confers resistance to tomato yellow leaf curl virus (TYLCV) and other geminiviruses. Tomato lines bearing *Ty-1* not only produce relatively higher amounts of viral small interfering (vsi)RNAs, but viral DNA also exhibits a higher amount of cytosine methylation. Whether Ty-1 specifically enhances posttranscriptional gene silencing (PTGS), leading to a degradation of RNA target molecules and primarily relying on 21–22 nucleotides (nts) siRNAs, and/or transcriptional gene silencing (TGS), leading to the methylation of cytosines within DNA target sequences and relying on 24-nts siRNAs, was unknown. In this study, small RNAs were isolated from systemically TYLCV-infected leaves of *Ty-1* encoding tomato plants and susceptible tomato Moneymaker (MM) and sequence analyzed. While in susceptible tomato plants vsiRNAs of the 21-nt size class were predominant, their amount was drastically reduced in tomato containing *Ty-1*. The latter, instead, revealed elevated levels of vsiRNAs of the 22- and 24-nt size classes. In addition, the genomic distribution profiles of the vsiRNAs were changed in *Ty-1* plants compared with those from susceptible MM. In MM three clear hotspots were seen, but these were less pronounced in *Ty-1* plants, likely due to enhanced transitive silencing to neighboring viral genomic sequences. The largest increase in the amount of vsiRNAs was observed in the intergenic region and the V1 viral gene. The results suggest that Ty-1 enhances an antiviral TGS response. Whether the elevated levels of 22 nts vsiRNAs contribute to an enhanced PTGS response or an additional TGS response involving a noncanonical pathway of RNA dependent DNA methylation remains to be investigated.

## Introduction

RNA interference (RNAi) is a highly conserved mechanism in eukaryotes involved in several processes like gene regulation, development, and silencing of transposable elements (Han et al., 2004; Ding and Voinnet, 2007; Erdmann and Picard, 2020). It also acts as an important defense mechanism against viruses in plants. The trigger for RNAi is double-stranded RNA (dsRNA), which can arise as replication intermediates, overlapping transcripts, or folded mRNA structures during viral infection (Ding and Voinnet, 2007). The dsRNA is recognized by DICER-LIKE proteins (DCL), RNAse III-type enzymes, that cleaves the dsRNA into small interfering RNA (siRNAs) of different size classes, ranging from 21–24 nucleotide (nt) in length (Hammond, 2005). From a 21-nt siRNA, one strand is loaded into an RNA-induced silencing complex (RISC) containing an Argonaute (AGO)1 core protein, which subsequently guides the search for complementary target RNA molecules. The latter either become cleaved by the slicer activity of AGO1 or become translational arrested (Mallory and Vaucheret, 2010), a pathway that is generally referred to as posttranscriptional gene silencing (PTGS). In contrast, one strand of 24-nt siRNA loads into a RISC complex containing AGO4 as the core component, which assists in the search for complementary target DNA sequences *via* an RNA scaffold. This leads to the methylation of cytosine residues within the target DNA sequence *via* recruitment of DNA methyltransferases (Law and Jacobsen, 2010; Mallory and Vaucheret, 2010; Duan et al., 2015), and this pathway is generally named transcriptional gene silencing (TGS). RNA viruses are targeted by the PTGS pathway only, while DNA viruses are prone to both PTGS and TGS.

The model plant *Arabidopsis thaliana* encodes four DCL and 10 AGO proteins (Bologna and Voinnet, 2014). The proteins not only act in different pathways and have specialized functions but are also found to be function-redundant. DCL1 is involved in the production of microRNAs (miRNAs) from (host-encoded) miRNA precursors, which regulate the expression of genes (Zeng et al., 2003; Han et al., 2004). DCL2 and DCL4 produce siRNAs of 22-nt and 21-nt in size respectively, which both function in the PTGS pathway, while DCL3 produces 24-nt siRNAs that are involved in TGS (Bologna and Voinnet, 2014). The canonical pathway leading to RNA-dependent DNA methylation (RdDM) is mostly studied in the silencing of transposable elements, and less in the context of antiviral defense against DNA viruses (Zhang et al., 2018; Kenchanmane Raju et al., 2019; Erdmann and Picard, 2020). Recently, alternative, noncanonical pathways leading to RdDM are found which make use of elements of the PTGS machinery, and one pathway which is DCL independent and produces siRNAs *via* the action of 3′ to 5′ exonuclease (Marí-Ordóñez et al., 2013; McCue et al., 2015; Cuerda-Gil and Slotkin, 2016; Ye et al., 2016; Kenchanmane Raju et al., 2019; Erdmann and Picard, 2020).

In plants, the RNAi signal is amplified by RNA-dependent-RNA polymerases (RDRs) (Yu et al., 2003; Wang et al., 2010). *A. thaliana* codes for six RDRs, named RDR1 to RDR6. RDR1, 2, and 6 are the best characterized with an established role in RNAi (Willmann et al., 2011), and belong to a so-called alpha-class due to the presence of a highly conserved core motif. RDR6 converts aberrant RNA, resulting from DCL cleavage of a primary target RNA molecule or transgene expression, in dsRNA. The subsequent processing by DCLs leads to the production of a pool of secondary siRNAs (Wang et al., 2010), which contributes to a strong antiviral response. Plants in which RDR6 has been knocked out generally become hypersusceptible to RNA viruses (Schwach et al., 2005; Donaire et al., 2008). RDR activity also leads to the spreading of the silencing signal into sequences neighboring the initially targeted sequence, a process called transitivity (Parent et al., 2015; de Felippes and Waterhouse, 2020). Like RDR6, RDR1 enhances the antiviral PTGS pathway, but its function is less understood (Donaire et al., 2008; Qi et al., 2009; Garcia-Ruiz et al., 2010). RDR2 plays an important role in the TGS pathway and has been most well studied in relation to the silencing of transposable elements. It produces dsRNA of RNA polymerase IV transcripts, which are subsequently processed by DCL3 into 24 nt siRNAs (Blevins et al., 2015). RDR1 and RDR6 have also been implicated in a noncanonical RdDM pathway since mutation of those RDRs results in decreased DNA methylation (Pontier et al., 2012; Stroud et al., 2013).

Tomato yellow leaf curl virus (TYLCV) is a member of the *Geminiviridae* family and representative of the whitefly *Bemisia tabaci*-transmitted begomoviruses. TYLCV contains a circular single-stranded DNA genome of ~2.7 kb that replicates in the nucleus and codes for six proteins, two in virion sense strand and four in the complementary sense strand (Zerbini et al., 2017). Between the sense and complimentary sense open reading frames (ORFs), the intergenic region (IR) is located which is necessary for viral replication and functions as a promoter for transcription of viral genes (Borah et al., 2016). TYLCV is prone to antiviral PTGS and TGS, and during infection, 21-, 22-, and 24-nt viral small interfering RNAs (vsiRNAs) complementary to the viral genome are produced (Piedra-Aguilera et al., 2019). The initial dsRNA trigger is thought to be secondary folding structures in mRNA and/or overlapping transcripts of the sense and complementary sense ORFs (Ramesh et al., 2017). TGS is assumed to be more important than PTGS in the antiviral defense against geminiviruses since viral DNA in recovered tissue is hypermethylated and is associated with inactive chromatin markers (Ceniceros-Ojeda et al., 2016). In addition, plants deficient in elements of the RdDM pathway become hypersusceptible (Raja et al., 2008, 2014; Jackel et al., 2016). Interestingly, RDR2 knock-out plants are not hypersusceptible against geminiviruses and vsiRNA profiles against the geminivirus cabbage leaf curl virus (CaLCuV) are not remarkably changed in RDR1,2,6 triple knock out plants compared with wild type plants (Raja et al., 2008; Aregger et al., 2012). This suggests that RDR 1, 2, and 6 are not involved in the antiviral RNAi response against geminiviruses, and other RDRs may be implicated.

The cloning and identification of the *Ty-1* dominant resistance gene from *Solanum chilense* against TYLCV unveiled a new class of (non-NBS-LRR) resistance genes (Verlaan et al., 2013). The gene encodes an RDR from the gamma class, to which RDR3, 4, and 5 belong, and is distinct from RDR1, 2, and 6 of the alpha class (Wassenegger and Krczal, 2006). Until that moment, no function was assigned to any of the RDRs from the gamma class. Transgenic *Nicotiana benthamiana* and *Solanum lycopersicum* plants expressing Ty-1 showed resistance to several geminiviruses similar to the *Ty-1* introgression line, showing that Ty-1, as RDR, is solely responsible for this resistance (Voorburg et al., 2020). Other studies revealed that Ty-1 enhances the antiviral RNAi response, as inferred from elevated levels of vsiRNAs and increased methylation of cytosine residues in the viral DNA genome in *Ty-1* bearing tomato (Butterbach et al., 2014). To further elucidate the role of Ty-1 in the biogenesis of vsiRNAs and the antiviral response to TYLCV, in this study small RNAs (sRNAs) were purified from susceptible and resistant *Ty-1* bearing tomato plants. Sequence analysis and the genomic distribution of vsiRNAs revealed enhanced transitive silencing and an interesting increase in the production of 22 nt vsiRNAs.

## Materials and Methods

### Plant Material and Virus Stock

During this study, *S. lycopersicum* plants were maintained in a greenhouse at 23°C during the day and at 21°C at night (16 h light/8 h dark regime) and relative humidity of 60%. *S. lycopersicum* cv. Moneymaker (MM) was used as susceptible control and a *Ty-1* introgression line was derived from *S. chilense* LA1969 (Verlaan et al., 2013). Plants were infected with TYLCV *via* agroinoculation with an infectious clone of the TYLCV Israel strain isolated from Almeria, Spain, as described by Morilla et al. (2005) (GenBank AJ489258.1) or with untransformed agrobacteria as control (strain LBA4404).

### Agroinoculation

The protocol as described in Voorburg et al. (2020) was followed to perform the agrobacterium transient transformation assays, which is a slightly modified protocol of Bucher et al. (2003). In brief, *A. tumefaciens* was grown overnight at 28°C in 3 ml LB3 (10 gl^−1^ trypton, 5 gl^−1^ yeast, 4 gl^−1^ NaCl, 1 gl^−1^ KCl, 3 gl^−1^ MgSO_4_·2H_2_O) medium containing proper antibiotic selection pressure. From this culture, 600 μl was incubated overnight in 3 ml induction medium (10.5 gl^−1^ K_2_HPO_4_, 4.5 gl^−1^ KH_2_PO_4_, 1 gl^−1^ (NH_4_)_2_SO_4_, 0.5 gl^−1^ sodium citrate. 2H_2_O, 1 mM MgSO_4_·7H_2_O, 0.2% (w/v) glucose, 0.5% (v/v) glycerol, 50 μM acetosyringone, 10 mM 2-(N-Morpholino) ethanesulfonic acid [(MES), pH 5.6)]. The next day bacteria were pelleted by centrifugation (15 min. at 2,670 *g*) and resuspended in MS MES buffer [Murashige and Skoog medium (Duchefa biochemie) supplemented with 150 μM acetosyringone, 10 mM MES and 87 mM sucrose] at an OD600 of 0.5. The abaxial side of the first two true leaves of the 3-week old tomato seedlings was infiltrated with the agrobacteria by pressure inoculation with a needle-less syringe.

### Nucleic Acid Purification

First, systemically infected leaves (top leaves) were snap-frozen in liquid nitrogen and stored at −80°C. RNA was isolated from this tissue using the mirVana miRNA isolation kit (Life Technologies) according to the protocol of manufacturers. In brief, upon grinding the leaf tissue in liquid nitrogen, the cells were lysed by adding lysis/binding buffer in a 1:10 ratio (w/v) and mixing thoroughly. Subsequently, 1/10 volume of miRNA homogenate additive was added, followed by a 10-min incubation on ice. RNA was extracted by adding acid-phenol: chloroform, mixing and spinning the sample. The aqueous phase was transferred to a new tube and 1/3 volume of 100% ethanol was added. Upon mixing thoroughly, the sample was passed through a Filter Cartridge. From this point, both the filtrate, containing the sRNAs, as the filter cartridge, containing the RNA fraction depleted of sRNAs, was further processed. To the filtrate 2/3 volume, 100% ethanol was added and this mixture was passed through a second filter cartridge. This filter cartridge was washed once with miRNA wash solution 1 and subsequently washed two times with wash solution 2/3. The sRNA fraction was eluted with MiliQ. The first filter cartridge containing the RNA fraction depleted of sRNAs was washed once with miRNA wash solution 1 and subsequently washed two times with wash solution 2/3. This RNA fraction was also eluted with MiliQ. The integrity of the RNA fractions was checked on an RNAse free agarose gel. From the samples used for the RNA isolations, also DNA was isolated following the cetyltrimethyl ammonium bromide (CTAB) method of Doyle and Doyle (1987) with slight modifications as described by Doyle and Doyle (1987), Fulton et al. (1995), and Voorburg et al. (2020). In brief, grinded plant material was mixed with CTAB buffer (0.1 M TRIS, 0.7 M NaCl, 0.01 M EDTA, 2% CTAB) and subsequently incubated for 1 h at 65°C. After chloroform isoamyl alcohol extraction, the DNA was precipitated from the aqueous phase by adding isopropanol in a 1:1 ratio. DNA was pelleted, dried, and dissolved in MilliQ. DNA and RNA concentrations were measured with a Nanodrop ND-1000 device.

### Library Preparation and Sequencing

The sRNA fraction was analyzed by capillary electrophoresis on a Shimadzu MultiNA microchip. The QIAseq miRNA library kit (Qiagen) was used according to the manufacturer's instructions to synthesize cDNA. In short, oligonucleotide adapters were ligated to the 5′ and 3′ ends of the RNA samples. First-strand cDNA was synthesized using M-MLV reverse transcriptase and the 3′ adapter as a primer. Amplification of the resulting cDNA was performed by PCR with a high-fidelity DNA polymerase in 16 PCR cycles. The 3′ sequencing adapters included a sample specific six nucleotide long barcode sequence. The cDNA was purified using the magnetic beads provided in the kit. The cDNA was analyzed by capillary electrophoresis on a Shimadzu MultiNA microchip. The cDNA samples were pooled in approximately equimolar amounts and the size range of 160–200 bp was selected from a polyacrylamide gel. An aliquot of the size fractionated pool was analyzed by capillary electrophoresis on a Shimadzu MultiNA microchip. The cDNA pool was sequenced on an Illumina NextSeq 500 system using a 75 bp read length. The original contributions presented in the study are publicly available. This data can be found here: https://www.ncbi.nlm.nih.gov/sra/PRJNA764425/.

### Bioinformatics Analysis of sRNA Data

The data analysis was performed on the Galaxy server (Afgan et al., 2018). Quality assessments were performed with the FastQC tool (Galaxy version 0.72+galaxy1). From each read, the adapter sequences were removed using the clip tool (Galaxy Version 1.0.3+galaxy0). Sequence length was determined by using the compute sequence length tool (Galaxy Version 1.0.3) and the count tool (Galaxy Version 1.0.3). Reads were mapped to the tomato genome (solgenomics, SL4.0) using Bowtie2 (Galaxy Version 2.3.4.3+galaxy0, default settings), and read length of tomato aligned reads calculated with the compute sequence length tool (Galaxy Version 1.0.3) and the count tool (Galaxy Version 1.0.3). Reads were mapped to the viral genome (TYLCV, GenBank AJ489258.1) using Bowtie2 (Galaxy Version 2.3.4.3+galaxy0, allowing 1 mismatch). From the bowtie output, genome profiles were generated by using the count (Galaxy Version 1.0.3), filter (Galaxy Version 1.1.1), join two datasets (Galaxy Version 2.1.3), compute (Galaxy Version 1.3.1), and advanced cut (Galaxy Version 1.1.0) tools. To determine the 5′ end nucleotides, the sequence logo tool (Galaxy Version 3.5.0) was used.

### qPCR for Titer Determination

Relative virus titers of TYLCV were determined by qPCR using actin (Solyc04g011500) as the internal control. The reaction mixture contained 1× SybrSelect (Applied Biosystems), 300 nM forward primer, 300 nM reverse primer, and 10 ng genomic DNA. The primers TYLCV-F (5′-TTCGTCTAGATATTCCCTATATGAGGAGGTA-3′) and TYLCV-R (5′-GGGAAGCCCATTCAAATTAAAGG-3′) were used to amplify TYLCV, and actin-F (5′-GAAATAGCATAAGATGGCAGACG-3′) and actin-R (5′-ATACCCACCATCACACCAGTAT-3′) to amplify actin (Powell et al., 2012; Verlaan et al., 2013). The qPCR was performed in an Applied Biosystems AB7500, using the following cycling conditions: 2 min 95°C, 40 cycles of 15 s 95°C and 1 min 60°C, followed by a melting curve with 0.5°C steps from 60 to 95°C to determine PCR specificity. Relative viral titers were calculated using the DeltaDeltaCt method (Livak and Schmittgen, 2001). Values were normalized relative to the internal control and calibrated to levels of the TYLCV infected MM plants, which were set as 1.

## Results

### Sample Preparation for vsiRNA Profiling

**Figure 1:**
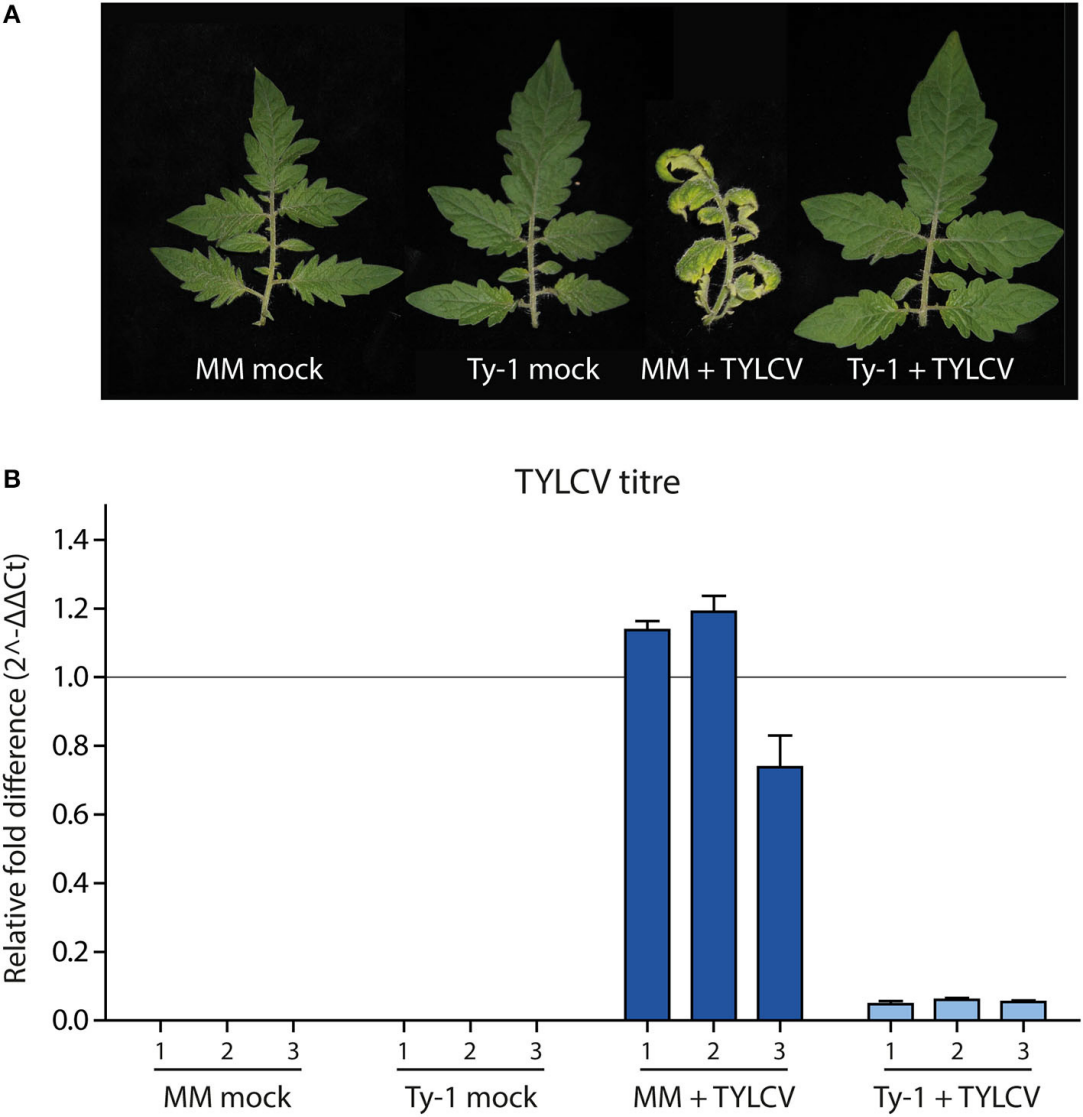
Symptoms and viral titers in MM and *Ty-1* bearing tomato. **(A)** Symptoms upon infection with TYLCV or mock infection in respectively *Ty-1* introgression tomato and susceptible MM at 29 days post infection. **(B)** Quantification of TYLCV titer in MM and the *Ty-1* introgression line. Values were normalized to actin and calibrated to the levels in MM plants (set to 1). Every bar represents the average viral titer and standard deviation based on technical replicates of individual plants.

Tomato lines containing *Ty-1* were previously shown to contain increased amounts of vsiRNAs during TYLCV infection compared to susceptible MM plants. However, nothing was known yet on the exact role of Ty-1 in the biogenesis of vsiRNAs, i.e., on the size distribution of vsiRNAs and their genomic distribution profile. To determine the effect of the resistance gene *Ty-1* on vsiRNA profiles in more detail, sRNAs were purified from infected and uninfected susceptible and *Ty-1* encoding tomato plants and then analyzed *via* deep sequencing. The used *Ty-1* bearing tomato plants are an introgression line, and it was previously shown that silencing of *Ty-1* breaks the resistance, ruling out the involvement of another resistance gene (Verlaan et al., 2013). First, five 3-week old *Ty-1* bearing tomato seedlings and susceptible tomato MM seedlings were either infected with TYLCV by agroinoculation or, as the negative control, infiltrated with untransformed agrobacteria. As expected, during the progression of the disease MM plants infected with TYLCV showed clear yellowing and curling of the top leaves (Figure 1A), while infected *Ty-1* plants did not show any symptoms, just like uninfected control plants. At 29 days postinfection (dpi) systemic leaves were harvested. From each group, three plants were used to collect three independent biological replicates. To confirm a successful infection, DNA was extracted and virus titers determined *via* qPCR. The fold difference in titer was calculated using the DeltaDeltaCt method (Livak and Schmittgen, 2001) (Figure 1B). While viral titers in infected MM plants were high, those in the *Ty-1* introgression line were reduced ~20-fold. No virus was detected in the mock control plants, which all were in accordance with previous results (Voorburg et al., 2020).

### Identification of vsiRNA Populations in MM and *Ty-1* Plants

**Figure 2:**
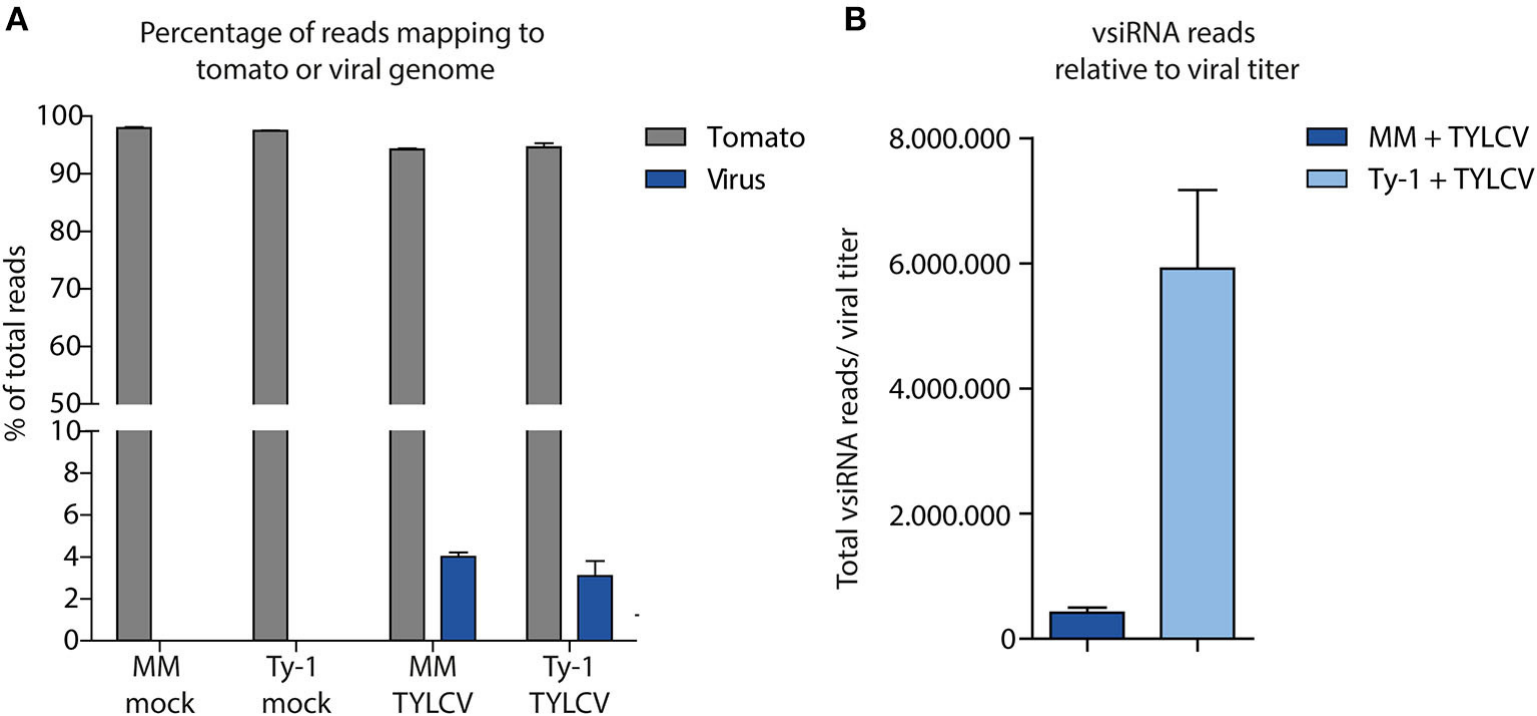
Distribution of sRNAs mapping to the tomato genome or viral genome. **(A)** sRNAs were isolated from mock-treated and TYLCV-infected susceptible MM and *Ty-1* bearing tomato and subsequently sequenced. The sRNAs were mapped to either the tomato genome or the viral genome using Bowtie2. The percentage of reads mapping to either the tomato or TYLCV genome relative to the total reads is depicted. Each bar represents the mean percentage and standard deviation of three biological replicates. **(B)** The number of sRNAs mapped to the viral genome was corrected for the relative amount of virus found in the samples according to qPCR. The bars represent the mean and standard deviation of three biological replicates.

After having verified viral titers in the mock treated and virus-infected MM and *Ty-1* tomato introgression line, systemic leaves collected from each treatment were used for the purification of sRNAs and subsequent library preparation. Deep sequencing of the sRNAs from three biological replicates of each treatment resulted in libraries containing in total between 9.5 and 11.8 million clean reads, varying in length from 15–70 nts (Table 1). The libraries of mock infected samples consisted almost fully of reads mapping to the tomato genome (average 97.7%) and with 0% of the reads mapping to the viral genome (Figure 2A, Table 1). siRNAs mapping to the viral genome (vsiRNAs) in samples of TYLCV infected plants represented on average 4.0% and 3.1% of total reads in MM and *Ty-1* plants, respectively (Figure 2A). The amount of viral reads was comparable between the MM and *Ty-1* plants, while previously an increased amount of vsiRNAs in *Ty-1* plants compared to MM was reported (Butterbach et al., 2014). However, it is important to note the lower viral titer determined in *Ty-1* plants. When the ratio of viral reads relative to the viral titer was determined, a strong increase in vsiRNAs per viral genome was observed in *Ty-1* plants (Table 1, Figure 2B).

**Table 1:** Overview of the sRNA libraries.

**Sample**	**Total clean reads**	**Reads mapped to Tomato**	**Reads mapped to TYLCV**	**Viral titer (average titer MM+ TYLCV set to 1)**	**TYLCV reads relative to titer (reads/viral titer)**
MM Mock 1	10,297,567	10,082,269 (97.91%)	141 (0.00%)	0.00	
MM Mock 2	11,218,593	10,982,695 (97.90%)	60 (0.00%)	0.00	
MM Mock 3	10,217,804	10,024,767 (98.11%)	92 (0.00%)	0.00	
*Ty-1* Mock 1	11,840,147	11,537,941 (97.45%)	45 (0.00%)	0.00	
*Ty-1* Mock 2	10,767,517	10,486,410 (97.39%)	220 (0.00%)	0.00	
*Ty-1* Mock 3	10,992,886	10,724,901 (97.56%)	23 (0.00%)	0.00	
MM + TYLCV 1	10,211,664	9,631,616 (94.32%)	422,028 (4.13%)	1.14	370,956
MM + TYLCV 2	10,683,014	10,049,426 (94.07%)	441,441 (4.13%)	1.19	370,620
MM + TYLCV 3	10,048,791	9,482,625 (94.37%)	381,275 (3.79%)	0.74	516,655
*Ty-1* + TYLCV 1	11,453,220	10,907,151 (95.23%)	283,914 (2.48%)	0.05	6,022,338
*Ty-1* + TYLCV 2	9,553,480	9,048,851 (94.72%)	279,112 (2.92%)	0.06	4,611,120
*Ty-1* + TYLCV 3	9,893,962	9,283,591 (93.83%)	384,052 (3.88%)	0.05	7,115,461

*MM or Ty-1 bearing tomato plants were infected with TYLCV and 29 days post infection, sRNAs were isolated from systemic leaves and sequenced. Data from three biological replicates are shown. The amount of raw reads and the amount of reads mapped to either the tomato or TYLCV genome using Bowtie2 are depicted*.

### Increased vsiRNAs of 22- and 24-nt Size Classes in *Ty-1* Plants

**Figure 3:**
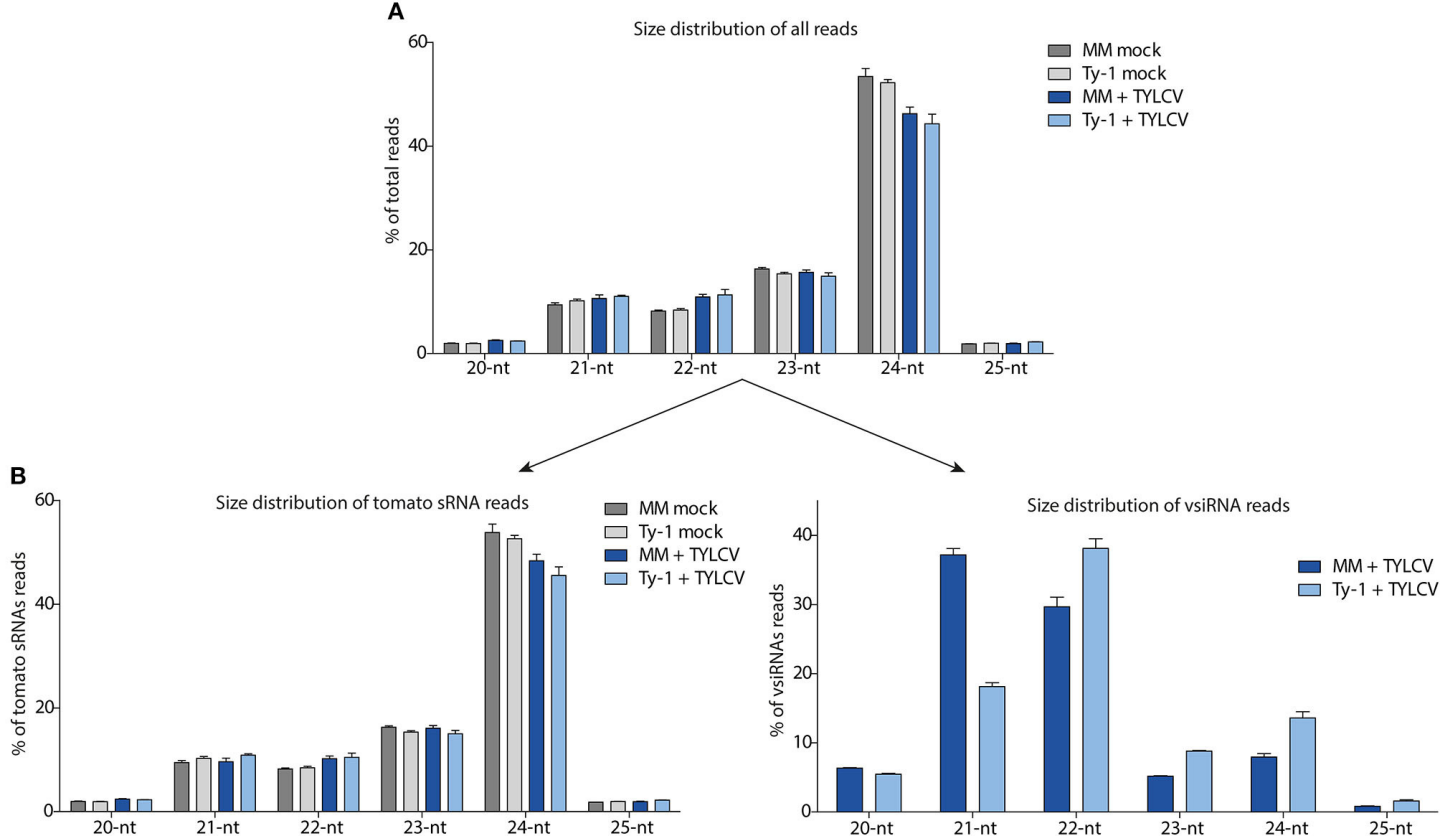
Size distribution of the total sRNAs, sRNAs mapping to the tomato genome, and siRNAs mapping to the viral genome (vsiRNAs). The sRNAs reads of the four groups (MM and *Ty-1* tomato, mocked treated, and TYLCV-infected) were analyzed on size. **(A)** The percentage for each size class (20–25 nt) on the total amount of reads is plotted. **(B)** The library of all reads was split into host-derived sRNAs and vsiRNAs and the percentage for each size class (20–25 nt) is depicted relative to the total amount of reads mapping to the tomato genome (left panel), and relative to the total amount of reads mapping to the viral genome (right panel). Viral reads are only analyzed from TYLCV infected plants (right panel).

To further characterize the sRNAs, the size distribution was determined within the total pool of sRNAs. The most abundant sRNAs, as observed in samples from all treatments, were from the 24-nt size class, covering about 42–54% of all sRNA reads (Figure 3A). The sRNA reads were next split into those sRNAs mapping to the tomato genome and those mapping to the TYLCV genome (vsiRNAs). Also, among the tomato specific sRNAs, the 24-nt size class was by far the most prevalent, representing 54% of all sRNAs in uninfected MM, 53% in uninfected *Ty-1* plants, 48% in TYLCV-infected MM plants, and 46% in TYLCV infected *Ty-1* plants (Figure 3B). Besides the 24-nt sRNAs, the other most frequently found sRNAs were from 23-, 22-, and 21-nt size classes, which were all present in relative comparable amounts (8–16%) (Figure 3B). vsiRNAs were only collected from the TYLCV infected leaf samples and not found, as expected, in the mock infected plants. The most predominant class of vsiRNAs collected from susceptible MM, were from 21-nt (37%), followed by 22-nt (30%) and the less abundant classes of 24-nt (8%) and 23-nt (5%) (Figure 3B). In *Ty-1* plants, this pattern was changed, with the largest size class presented by 22-nt (38%), followed by 21-nt (18%), 24-nt (14%), and 23-nt (9%) (Figure 3B). So, the class of the 21-nt vsiRNAs was reduced by ~50% in *Ty-1* tomato compared with the susceptible MM, whereas the 22-, 23-, and 24-nt size classes were clearly increased (~25% increase for 22-nt and ~75% increase for 23- and 24-nt vsiRNAs).

**Figure 4:**
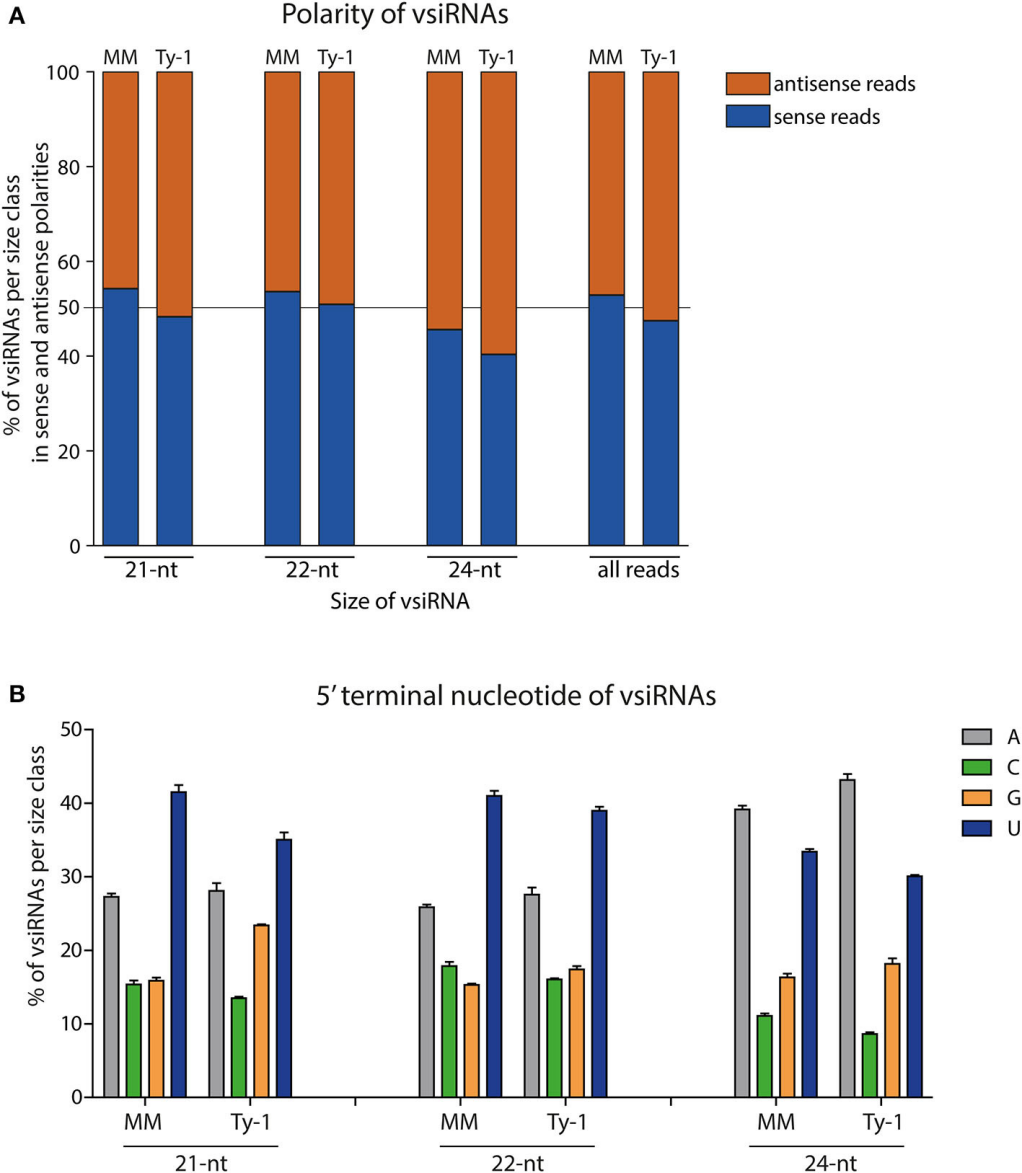
The polarity and 5′ terminal nucleotides of vsiRNA reads. **(A)** Viral siRNAs from TYLCV-infected MM or *Ty-1* bearing tomato depicted per size class (21-, 22-, 24-nt or all reads) and showing the percentage of sense and antisense oriented reads. The average of three biological replicates is depicted. **(B)** siRNAs from TYLCV-infected MM or *Ty-1* tomato depicted per size class of 21-, 22-, and 24-nt and showing the percentage of reads containing adenine (A), cytosine (C), guanine (G), or uracil (U) as 5′ terminal nucleotide. The bar represents the average and standard deviation of three biological replicates.

Having determined the size distribution of the vsiRNAs, the polarity of the reads was analyzed. This was done for the total viral reads, and the 21-, 22-, and 24-nt size classes. In MM plants, the majority of all viral reads (53%) and those of the size class 21-nt (54%) and 22-nt (54%) were in sense polarity (clockwise orientation of the viral genome), whereas of the 24-nt size class 46% was in sense polarity (Figure 4A). In *Ty-1* tomato, the amount of sense reads showed a small reduction of 3–6% in all the studied size classes when compared to MM plants, with 47% of all viral reads, 48% of 21-nt reads 51% of 22-nt reads, and 40% of 24-nt reads being in sense polarity (Figure 4A).

In light of the importance of the 5′ terminal nucleotide of siRNAs toward loading into different AGO proteins (Kim, 2008; Mi et al., 2008), the 5′ terminal nucleotide of viral reads was analyzed. In both MM and *Ty-1* tomato reads from the 21-nt and 22-nt classes were enriched in a 5′ terminal uridine (U), with a prevalence of around 40% in both groups. The second most dominant 5′ terminal nucleotide was adenosine (A), with an average prevalence of around 27% in both groups (Figure 4B). Within the 24-nt reads, A was observed most prevalent (~40%), followed by U (~30%) (Figure 4B). The same patterns were observed when reads of viral sense and antisense polarity were separately analyzed (Supplementary Figure S1), with the exception of two cases. Within the 21-nt and 22-nt reads of antisense polarity U and A were more equally presented at the 5′ end, whereas U was dominant within the sense reads. The 24-nt reads of sense polarity showed a comparable proportion of U and A as 5′ terminal nucleotide, while A was more dominant in the reads of antisense polarity (Supplementary Figure S1). No clear differences were observed in 5′ terminal nucleotides between vsiRNAs isolated from MM and *Ty-1* plants.

### TYLCV Is Targeted by vsiRNAs Covering the Entire Viral Genome in MM and *Ty-1* Plants

**Figure 5:**
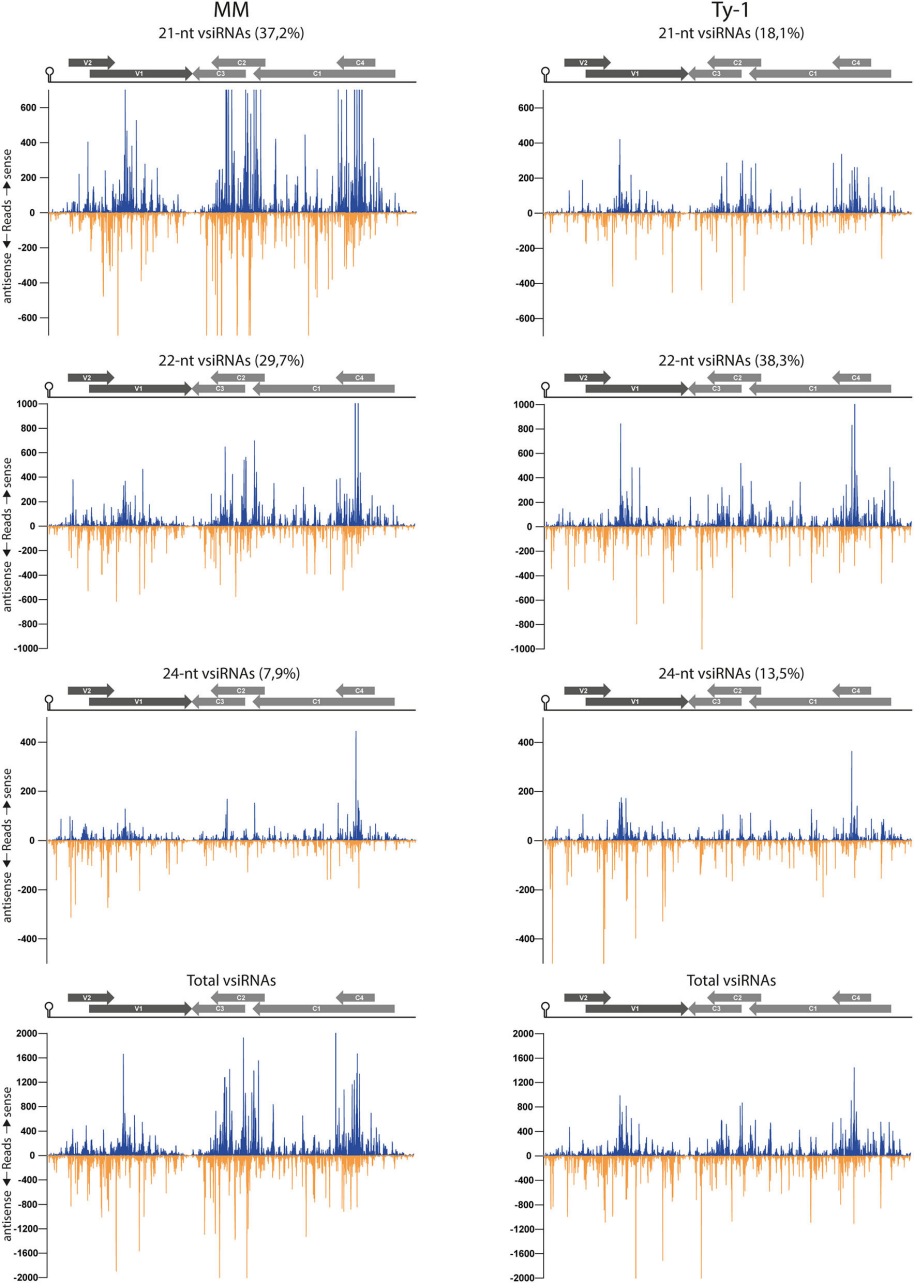
Distribution of vsiRNAs on the TYLCV genome in MM and *Ty-1* tomato. Viral siRNAs isolated from infected MM (left panel) or *Ty-1* (right panel) tomato were mapped on the genome of tomato yellow leaf curl virus (TYLCV). The number of reads at each nucleotide position of the TYLCV genome is plotted (average ofthree biological replicates), for either 21-, 22-, and 24-nt, and all vsiRNAs. Blue bars represent sense reads starting at each respective nucleotide, while the orange bars represent antisense reads ending at that position. The depicted percentage in the title represents the proportion that belongs to the size class relative to all vsiRNAs in either MM or *Ty-1* tomato. A schematic overview of the TYLCV genome is depicted above the graphs, with the viral ORFs indicated as gray arrows and the intergenic region at the left and right sides.

To examine the genomic distribution of vsiRNAs produced and analyze for possible changes in the presence of *Ty-1*, the reads derived from infected susceptible MM and resistant *Ty-1* tomato were plotted against the TYLCV genome. The results showed that in both MM and *Ty-1* plants the vsiRNAs cover the entire genome in viral sense and antisense orientation (Figure 5). For both MM and *Ty-1* plants, the genomic distribution was somewhat heterogenous, with several regions being covered by more and others by less vsiRNAs. This was reflected in all vsiRNA size classes (Figure 5) and observed highly consistent in all three biological replicates (Supplementary Figure S2). Hotspots, regions with a higher density of vsiRNAs per nucleotide, were visible in the viral genome distribution of both MM and *Ty-1* tomato, but these hotspots appeared more distinct in MM. Hotspots coincided with regions of overlapping ORFs, i.e., V1/V2, C1/C2/C3, and C1/C4 respectively (Figure 5, Table 2A), and were present in both the viral sense and antisense orientation and observed for all vsiRNA size classes. Only in the profile of 24-nt vsiRNAs, the hotspot at the C1/C2/C3 overlapping region was less pronounced.

**Table 2:** Distribution of vsiRNAs on the TYLCV genome.

**(A)**	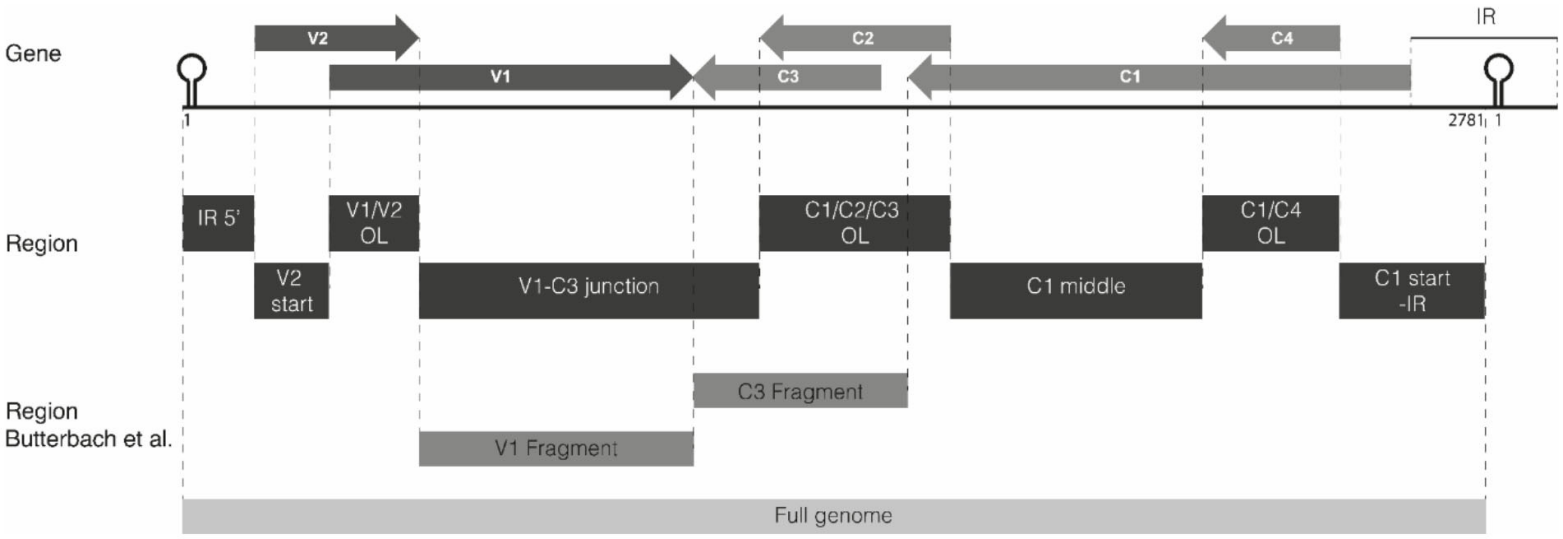						
**Gene**	**vsiRNAs (reads)**		**vsiRNA reads/nct**		**siRNA reads per nct/average vsiRNAs per nct (>1 is more siRNAs per nt than on average over full genome)**		**Change in *Ty-1* compared to MM**
	**MM**	***Ty-1***	**MM**	***Ty-1***	**MM**	***Ty-1***	***Ty-1*** **/MM**
V1	78,469	69,531	101.0	**89.5**	0.90	1.13	**1.25**
V2	38,170	23,722	108.7	67.6	0.97	0.85	0.88
C1	131,224	85,935	**122.2**	**80.0**	1.09	1.01	0.92
C2	91,725	41,039	**224.8**	**100.6**	2.01	1.27	0.63
C3	64,122	36,601	**158.3**	**90.4**	1.42	1.14	0.80
C4	56,298	33,759	**191.5**	**114.8**	1.72	1.45	0.84
IR	6,398	13,679	20.4	43.7	0.18	0.55	**3.01**
**Region**							
IR 5′	3,702	6,780	24.0	44.0	0.22	0.56	**2.58**
V2 start	14,090	8,051	88.1	50.3	0.79	0.63	0.80
V1/V2 OL	24,080	15,671	**126.1**	**82.0**	**1.13**	**1.04**	0.92
V1-C3 junction	61,472	63,886	84.6	**87.9**	0.76	**1.11**	**1.46**
C1/C2/C3 OL	91,725	41,039	**224.8**	**100.6**	**2.01**	**1.27**	0.63
C1 middle	48,577	32,755	90.5	61.0	0.81	0.77	0.95
C1/C4 OL	56,298	33,759	**191.5**	**114.8**	**1.72**	**1.45**	0.84
C1 start-IR	10,494	18,510	33.9	59.7	0.30	0.75	**2.48**
Full genome	310,437	220,450	111.6	79.3	1.00	1.00	
**(B) Regions corresponding to Butterbach et al. (2014)**							
	**vsiRNAs (reads)**		**Ratio reads v1/c3**				
	**MM**	***Ty-1***	**MM**	***Ty-1***			
V1 fragment	54,389	53,859	0.68	1.25			
C3 fragment	80,255	43,256					
**(C) Percentage Intergenic Region (IR) specific vsiRNAs of viral siRNAs of that size class**							
	**21nt**		**22nt**		**24nt**		
	**(+)**	**(–)**	**(+)**	**(–)**	**(+)**	**(–)**	
MM	0.53	0.66	0.93	1.32	1.78	3.64	
*Ty-1*	1.64	2.43	2.83	3.62	2.43	6.06	

*(A) MM or Ty-1 bearing tomatoes were infected with TYLCV and 29 days post infection, sRNAs were isolated from systemic leaves and sequenced. The average of three biological replicates is shown. On top, the viral genome is schematically depicted with different genes and genomic regions indicated by arrows and blocks, respectively. In the table, the number of vsiRNA reads of 21-, 22-, and 24-nt mapping to these genomic positions (as depicted in Figure 5) are shown. The density (vsiRNA reads/nucleotide) is calculated by dividing the amount of reads by the length of the genomic region or gene (column 4 for MM and column 5 for Ty-1). Column 6 (MM) and 7 (Ty-1) depict the same regions whether they have a higher or lower density than on average over the full genome. This is calculated by dividing the vsiRNA reads/nucleotide by the average amount of reads/nucleotide. In bold the regions with a higher density are indicated. In the last column, the change in Ty-1 compared to MM is depicted, with regions in bold that contain a higher density in Ty-1 compared to MM. (B) The number of reads (21-, 22-, and 24-nt) mapping to two fragments earlier studied on the amount of vsiRNAs by Butterbach et al. (2014) are indicated, and the ratio V1/C3 is calculated based on the amount of reads from this study. (C) The percentage of 21-, 22-, and 24-nt, respectively, mapping to the intergenic region (nucleotide 2623-154) of TYLCV in MM and Ty-1 bearing tomato. The depicted percentage represents the vsiRNA reads of each size class mapping to the Intergenic region in either viral sense (+) of antisense (–) orientation, relative to the total vsiRNA reads of that size class. The average of three biological replicates is shown*.

In both MM and *Ty-1* tomato, the intergenic region (IR) had the lowest abundance of vsiRNAs (Table 2A). Other regions with a low abundance in MM plants included the V2 start (5′ end of the V2 ORF until the start of V1 ORF), the V1–C3 junction (where the 3′ ends of the V1 and C3 transcripts are assumed to overlap), and the middle part of C1 (between the end of C4 and the start of C2) (Figure 5, Table 2A).

Not only the distribution of vsiRNA in all size classes was changed in the presence of Ty-1, the amount of reads per size class in MM and *Ty-1* plants was also changed (Figure 3B). This complicated the comparison of the genome distribution between the two groups. Therefore, per size class, the percentage of mapped vsiRNAs per nucleotide was plotted (Supplementary Figure S3). The three hotspots earlier observed at the regions with overlapping ORFs were again visible. However, when the proportion of vsiRNAs within the size class 21, 22, and 24-nts mapping to these hotspots was calculated, a higher amount was obtained for MM (55%) compared with *Ty-1* tomato (41%), indicating that in *Ty-1* tomato the hotspots were less targeted. To further quantify the observed differences in vsiRNA genome distribution between MM and *Ty-1* tomato, the number of reads/nucleotide was calculated for several genomic regions and compared with the reads/nucleotide in the situation of a completely homogenous distribution (Table 2A). The results strengthened the observation of less pronounced hotspots at the ORF overlapping regions of TYLCV in *Ty-1* tomato compared with the situation in MM. Interestingly, the region at the 3′-end of the V1 and C3 ORF (V1–C3 junction), which has a low abundance of vsiRNA in MM, showed to have a higher abundance than average in *Ty-1* plants (Table 2A, region “V1–C3 junction”). The increased density at V1 was in agreement with an earlier study, where the ratio of vsiRNAs mapping to V1/C3 changed in the presence of *Ty-1* (Butterbach et al., 2014). When the ratio was calculated for the same regions as used in the previous study, a comparable shift was observed (Table 2B).

**Figure 6:**
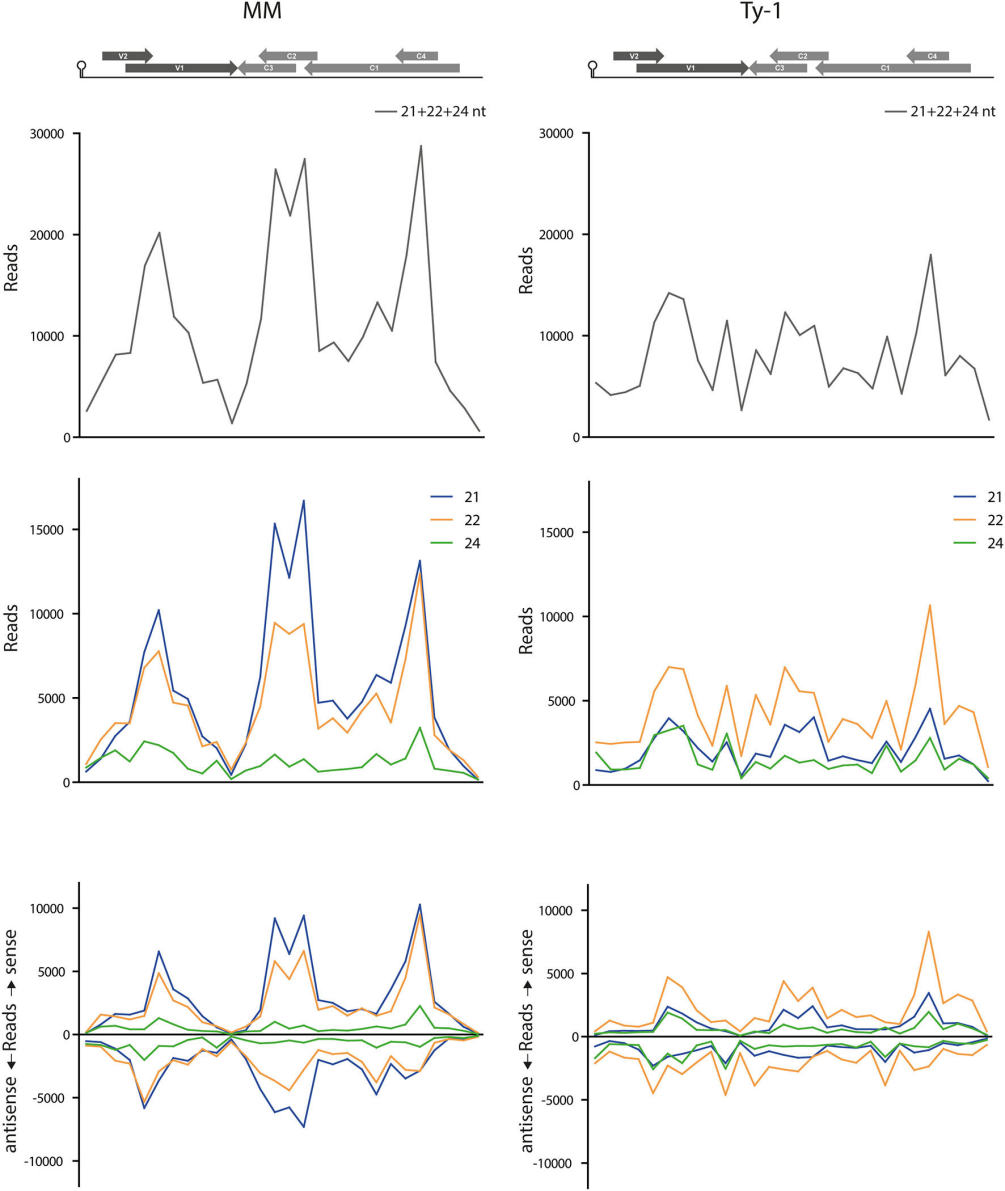
Amount of vsiRNA reads per 100 nucleotides in the TYLCV genome. Viral siRNAs isolated from infected MM (left panel) or *Ty-1* bearing (right panel) tomato are mapped on the genome of tomato yellow leaf curl virus (TYLCV). The numbers of reads per 100 nucleotides of the TYLCV genome (amount of reads for position 1–100, 101–200, etc.) are plotted. The graphs represent the average of three biological replicates. The upper panels present the amount of reads of 21-, 22-, and 24-nt size class together. The middle and lower panels depict the distribution of 21-, 22-, and 24-nt vsiRNA separately, either sense and antisense polarities taken together (middle panel), or both polarities specified (lower panel). A schematic overview of the TYLCV genome is presented at the top of the graph, with the viral ORFs indicated as gray arrows and the intergenic region at the left and right side.

To better visualize the change in genome distribution and obtain a broader overview image, the amount of reads per 100 nucleotides were plotted (Figure 6). Also, the percentage of mapped vsiRNAs per 100 nucleotides was plotted to compare the two groups without a possible bias by a difference in absolute reads (Supplementary Figure S4). Those plots confirmed the previous finding of the high density of vsiRNAs at the regions with the overlapping ORFs V1/V2 and C1/C4 in both MM and *Ty-1* plants. It also revealed that in *Ty-1* plants less vsiRNAs accumulated at the region with the overlapping ORFs C1/C2/C3, while this region is a clear hotspot in MM (Supplementary Figure S4; Table 2A). In addition, the amount of vsiRNAs that were mapped to the IR and V1 was increased in *Ty-1* plants, especially in an antisense orientation (Supplementary Figure S4; Table 2A).

### Targeting of the Intergenic Region of TYLCV

Comparison of the genomic distribution profiles of the vsiRNAs collected from susceptible MM and resistant *Ty-1* tomato indicated that the IR and the region “V1–C3 junction” were relatively more targeted in *Ty-1* tomato. The percentage of vsiRNAs to those regions was increased 3 and 1.5 fold, respectively (Table 2A). A previous study reported that in MM plants the IR was targeted relatively more by vsiRNAs of 24-nt than by those of 21-nt and 22-nt (Piedra-Aguilera et al., 2019). A closer look at the data from this study confirmed these observations (Table 2C). This study in addition showed that in *Ty-1* tomato, the amount of vsiRNAs mapping to the IR increased for all three size classes (21-, 22-, and 24-nt) in both polarities, but with the highest amounts of antisense vsiRNAs (Table 2C). In both MM and *Ty-1* tomato, the antisense reads of 24-nts vsiRNAs were most prevalent (3.6 and 6.1%, respectively, Table 2C).

**Figure 7:**
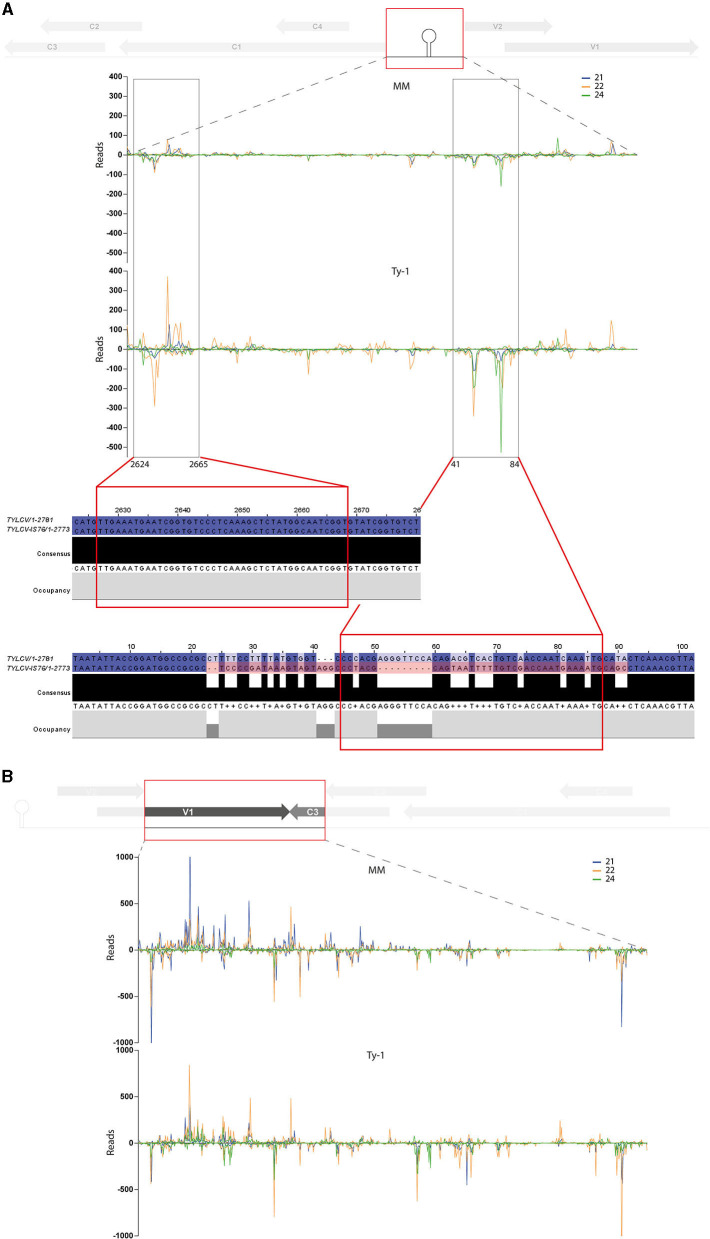
Close-up on the vsiRNA distribution in the intergenic region and “V1–C3 junction” and topological alignment of the recombinant IR region from TYLCV-IS76 with the vsiRNA of the intergenic region. Viral siRNAs isolated from infected MM (top panel) or *Ty-1* bearing (lower panel) tomato are mapped on the genome of tomato yellow leaf curl virus (TYLCV). The numbers of vsiRNA reads at each nucleotide position of the TYLCV genome are plotted (average of three biological replicates), for either 21-, 22-, and 24-nt vsiRNAs. Positive bars represent sense reads starting at each respective nucleotide, while the negative bars represent antisense reads ending at that position. A schematic overview of the TYLCV genome is depicted at the top of each graph, with the viral ORFs indicated as gray arrows and the region zoomed in boxed in red. **(A)** Genomic distribution of vsiRNAs in the intergenic region (nucleotides 2623-154). Regions of major differences between vsiRNA profiles of MM and *Ty-1* plants are highlighted in black boxes and underneath the nucleotide sequences of the corresponding regions from TYLCV and the *Ty-1* resistance-breaking TYLCV-IS76 are shown. **(B)** Genomic distribution of vsiRNAs in the region “V1–C3 junction” (nucleotides 506–1232).

To analyze whether the increased amount of vsiRNAs from the IR and “V1–C3 junction” region mapped to specific sequences (e.g., secondary structures) or were distributed comparable to the situation in MM, genomic distribution graphs were made zoomed in on these regions (Figure 7). In the region “V1–C3 junction” the increased amounts of vsiRNAs from *Ty-1* tomato distributed all over the region (Figure 7B). In contrast, vsiRNAs from the IR appeared to concentrate in two “sub-regions,” namely around nucleotides 2624–2665 and nucleotides 41–84 of the TYLCV genome (Figure 7A). Earlier, reports of resistance breaking strains in *Ty-1* tomato described recombination events between TYLCV and Tomato yellow leaf curl Sardinia virus (TYLCSV) taking place in the IR region (Belabess et al., 2015, 2016, 2018; Torre et al., 2018; Urbino et al., 2020), and with the smallest recombination event reported for the resistance breaking TYLCV-IS76 (Urbino et al., 2020). To analyze whether this region coincided with the hotspot “sub-regions” of the IR found in this study, the region of recombination in TYLCV-IS76 was compared with the sub-regions of increased vsiRNAs in *Ty-1* plants. The results showed that one region (nucleotide 41–84) indeed overlapped with the recombination event (Figure 7A).

### Potential Production of Dicer Independent siRNAs

Recently, an alternative Dicer-independent pathway resulting in genome methylation was discovered (Ye et al., 2016). In this process, longer RNA molecules bind to AGO4 and are subsequently trimmed, independent of Dicer, by 3′- to 5′-exonucleases into siRNAs. The population of siRNAs resulting from this shows up as a “ladder” pattern (~20–60 nt in size) and shares the same 5′ end but differ at their 3′ end with one nucleotide. Since not much is known yet on the role of members from the RDRγ in (viral) siRNA biogenesis, and whether these would assist in the production of dicer independent siRNAs (sidRNA), the reads collected from MM and *Ty-1* tomato were examined for molecules larger than 24-nt and matching features of sidRNAs. In both MM and *Ty-1* tomato TYLCV-specific vsiRNAs >24 nt were found, but the amounts of these were not substantially increased in the presence of *Ty-1* (Supplementary Figure S5A). Spots with potential sidRNAs were found by screening for vsiRNAs 25-33 nt in size and sharing the same 5′ end (for an example of a potential sidRNA refer to Supplementary Figure S5C). The potential sidRNA spots were mostly in antisense orientation and some were even consistently found in biological replicates, although the positions identified in *Ty-1* plants were different from those in MM plants (Supplementary Table S1, Supplementary Figures S5D–F). However, in general, their number was relatively low, i.e., 11 and 3 from MM and *Ty-1* plants, respectively (Supplementary Figure S5B).

## Discussion

The role of RDR1, 2, and 6, all three members of the RDRα class, in the biogenesis of siRNAs, already has been well established (Willmann et al., 2011). In this study, a first step was made to unravel the role of Ty-1, a dominant resistance gene protein against geminiviruses and member of the RDRγ class, in the biogenesis of siRNAs. To this end, susceptible MM and *Ty-1* containing tomatoes were either mock-infected or infected with TYLCV and comparatively analyzed on the biogenesis of vsiRNAs. Comparable amounts of vsiRNAs were observed in MM and *Ty-1* bearing plants, but the ratio of vsiRNAs to viral DNA copies in *Ty-1* tomato was increased about ~14-fold. In susceptible MM vsiRNAs of 21-nt were found to be dominant, whereas in *Ty-1* tomato vsiRNAs of 22-nt and 24-nt were increased and those of 21-nt were drastically reduced. Several vsiRNA hotspots were found, most of them mapping to genomic regions containing overlapping ORFs, but these hotspots were less pronounced in the presence of *Ty-1*. In *Ty-1* tomato, additionally more vsiRNAs were found to target the IR and the V1 ORF. Altogether, these results support earlier findings in which Ty-1 seems to strengthen an antiviral TGS response. Whether the increased production of 22-nt vsiRNAs also plays a role in this, involving a (noncanonical) RdDM pathway, distinct from the RDR2-mediated RdDM pathway, or whether these contribute to an antiviral PTGS response remains to be investigated.

The 5′ terminal nucleotide analysis revealed no difference between MM and *Ty-1* plants. The dominant 5′ terminal nucleotide of vsiRNAs of 21-nt and 22-nt size class was U, followed by A. For the 24-nt size class, the predominant 5′ terminal nucleotide was A, followed by U. Argonaute proteins have been shown to preferably associate with siRNAs of a certain size and 5′ terminal nucleotide (Mallory and Vaucheret, 2010). The findings in this study are in agreement with 5′ terminal nucleotide analyses of vsiRNAs as previously reported for TYLCV and other geminiviruses (Yang et al., 2011; Aregger et al., 2012; Piedra-Aguilera et al., 2019), and supported the notion that geminiviral siRNAs are loaded into several AGO proteins. Furthermore, the prevalent 5′U residue within the 22-nt vsiRNAs from *Ty-1* tomato, similar to the 5′ end residue prevalence of 22-nt vsiRNAs from susceptible MM, does not make a clear distinction on the involvement of a (new Ty-1 mediated) noncanonical RdDM pathway with AGO4/AGO6 or the PTGS pathway. Although the specificity of AGO4 and 6 is normally for 24-nt containing a 5′A and PTGS related siRNAs bound to AGO1 or AGO2 show a 5′U and 5′A bias respectively (Mi et al., 2008; Havecker et al., 2010), the AGO6 bound 22-nt siRNAs of the noncanonical pathway have less 5′ terminal nucleotide bias and contain both 5′A or 5′U (McCue et al., 2015). Determining the association of vsiRNAs to AGO proteins in MM and *Ty-1* plants during infection by performing a pull-down on several AGO proteins and subsequent sequencing, might shed light on the function of 22-nt vsiRNAs in TYLCV infected *Ty-1* tomato.

While in MM three hotspots were visible in the TYLCV genome at regions containing overlapping ORFs, they were less pronounced in the situation of *Ty-1* tomato, with the number of vsiRNA counts per nucleotide in these regions less deviating from the average amount of vsiRNAs per nucleotide over the full genome (Table 2). This was also seen for regions with a lower number of vsiRNAs (coldspots), indicating that the vsiRNAs are more equally spread over the genome in *Ty-1* tomato compared to MM. This is most likely explained by transitivity resulting from Ty-1. This process has earlier been described and starts with the conversion of (aberrant) single-stranded RNAs, resulting from primary RNA target cleavage, into dsRNAs by the action of RDRs. Their subsequent processing by DCLs leads to a pool of secondary siRNAs and the spreading of the siRNAs into the neighboring sequences from the initial target sequences, called transitivity (de Felippes and Waterhouse, 2020). An alternative explanation is that in *Ty-1* plants the expression pattern of viral genes might be changed, resulting in different amounts of templates to generate vsiRNAs.

An intriguing question that remains to be solved is in which pathway Ty-1 functions. Since geminiviruses seem to be most affected by TGS and the methylation of the TYLCV genome is enhanced in *Ty-1* tomato (Raja et al., 2008, 2014; Butterbach et al., 2014; Ceniceros-Ojeda et al., 2016; Jackel et al., 2016), it was hypothesized that Ty-1 amplifies the TGS response. Hence, an increase in the production of 24-nt vsiRNAs was expected. Interestingly, besides 24-nt also 22-nt vsiRNA levels were relatively increased, while those of 21-nt had dropped (Figure 3). Since 22-nt vsiRNAs are generally thought to function in the PTGS response and 24-nt vsiRNAs in the TGS response (Pumplin and Voinnet, 2013), the findings from this study would imply an increase of both pathways. However, alternative pathways leading to RdDM have been reported and are thought to be involved in the initiation of methylation (Kenchanmane Raju et al., 2019; Erdmann and Picard, 2020). Some of these noncanonical RdDM pathways involve 21–22 nt siRNAs and several elements of the PTGS pathway. In one pathway, RDR6-produced dsRNA is processed into 21–22 nt siRNAs by DCL2/DCL4. Instead of being loaded into AGO1, the siRNA is loaded into AGO6 and directs the RdDM (McCue et al., 2015). Alternatively, dsRNA produced by RDR6 becomes processed by DCL3 in 24-nt siRNAs and guides RdDM by AGO4 or AGO6 (Marí-Ordóñez et al., 2013). More recently, a Dicer-independent pathway has been described in which longer RNA molecules are bound by AGO4 and become trimmed by 3′ to 5′ exonucleases into sidRNAs which direct RdDM (Ye et al., 2016). In this study, potential sidRNAs targeting TYLCV were found in both MM and *Ty-1* plants, but their amounts were relatively low. To determine whether they present genuine sidRNAs, experiments with DCL knock-out plants are needed to show they are still produced in the absence of DCL. Still, these pathways are less well characterized than the canonical RdDM pathway [for some nice overviews on canonical and noncanonical RdDM readers are referred to Cuerda-Gil and Slotkin (2016), Kenchanmane Raju et al. (2019) and Erdmann and Picard (2020)]. Based on the data presented in this study, it is clear that Ty-1 enhances the production of 22 and 24-nt vsiRNAs, in which the 24 nts vsiRNAs are most likely involved in RdDM of the TYLCV genome. Whether the 22-nts vsiRNAs contribute to a stronger PTGS response and/or enhance a noncanonical RdDM pathway remains to be further investigated. Creating a knock-out or knock-down of DCL and AGO genes in *Ty-1* plants and measuring the effect on resistance could unravel which proteins are major players in the pathway of Ty-1, although redundancy of these elements will make this challenging. In addition, the cellular localization and interactions of Ty-1 might give further insights into the pathway.

The increase in 22-nt and 24-nt vsiRNAs in *Ty-1* plants is interesting in light of systemic silencing and tissue specificity of the virus. RNAi signals systemically spread *via* the phloem to other plant tissues, and both 22-nt and 24-nt sRNAs have been suggested to be able to move systemically (Molnar et al., 2010; Chen et al., 2018; Zhang et al., 2019). However, it remains debated which molecule is transported (Zhang et al., 2019). Considering TYLCV is a virus that is phloem-limited, i.e., replication primarily takes place there, it is tempting to speculate that vsiRNAs that are able to move systemically might be of higher importance to combat geminiviruses (Rojas et al., 2001; Morilla et al., 2004). It also raises an interesting question about the available RNAi machinery: Which elements of the RNAi machinery are expressed in the phloem tissue and is Ty-1 also localized there? The expression of RNAi elements is not equal in all tissues, e.g., different AGO proteins show distinct tissue specificity (Havecker et al., 2010). Not much is known on the composition of the RNAi machinery (e.g., DCLs, AGOs, RDRs) in phloem tissue. Studying the tissue specific expression of Ty-1 and other RNAi elements in phloem tissue could be a first step to unravel this. Phloem tissue consists of several specialized cell types like phloem parenchyma cells, companion cells, and sieve elements. The latter are elongated cells that interconnect into sieve tubes and present the conduits for phloem sap, but also for viruses and siRNAs to (systemically) disperse within a host. Although sieve elements are alive and contain organelles, RNAs and a proteome, they lack a nucleus and therefore may lack the RDR2-mediated TGS (canonical RdDM) pathway (Furuta et al., 2014). TYLCV DNA is mainly present in nuclei of phloem parenchymal and companion cells, although the DNA was also detected in the lumen of sieve tubes (Morilla et al., 2004). The sieve tube localization resembles most likely virions which are transported (Morilla et al., 2004), since the virus depends on the nuclear cellular DNA replication machinery to replicate (Saunders et al., 1992; Gutierrez, 2000). So, TGS activity will be found mainly in phloem parenchymal and companion cells. Whether this can move from neighboring companion cells to sieve tubes and can exert any action there is not known.

Although *Ty-1* presents an atypical (non-NBS-LRR) resistance gene and does not lead to viral clearance, i.e., low titers of virus can still be detected, TYLCV isolates that are able to “break” *Ty-1* resistance have been found. One of the best studied one is TYLCV-IS76, resulting from a recombination event within the IR between two parental strains, TYLCV and TYLCSV. Under experimental conditions, this recombinant showed to have a selective advantage compared to the parental viruses in *Ty-1* tomato (Belabess et al., 2016). Recently it was proposed that this selective advantage relates to intragenomic interactions since *in vitro* generated recombinants derived from other strains and virus recombination at a slightly different breakpoint (IS141) also exhibit a higher fitness than the parental viruses (Urbino et al., 2020). Within this study increased amounts of vsiRNAs were found in *Ty-1* tomato, that mapped to the region of the recombination event. However, this region did not exhibit an increased level of cytosine methylation during an earlier study (Butterbach et al., 2014). Whether and how the vsiRNAs mapping to this region plays a role in the resistance mechanism of *Ty-1* and whether antiviral targeting of this (important) region acts as a driver for recombination to generate resistance breaking strains like TYLCV-IS76 remains to be further investigated.

An earlier study has shown that *Ty-3* and *Ty-1* are allelic (Verlaan et al., 2013), and although it would be interesting to analyze the siRNA profile collected from TYLCV-infected *Ty-3* lines, it is likely going to be comparable to the one of *Ty-1*. Analyses of siRNA profiles from other TYLCV-resistant cultivars might be interesting as well, e.g., from those of which the resistance trait is not yet known, or of tomato plants bearing *Ty-2*. However, considering *Ty-2* encodes an NB-LRR and leads to a hypersensitive response, tomatoes containing *Ty-2* will likely contain less antiviral siRNAs (due to low replication levels), that otherwise will be comparable to susceptible cultivars. For this reason, siRNA profiles from *Ty-2* will not contribute to advanced insight into the *Ty-1*-mediated antiviral TGS response.

In conclusion, the data presented in this study show that Ty-1 changes the antiviral siRNA profile of TYLCV and supports the earlier observations that Ty-1 functions in enhancing the RNAi response. How and where Ty-1 functions to produce dsRNAs and which DLCs and AGOs are subsequently involved in the downstream RNAi will be one of the challenges for the future.

## Data Availability Statement

The datasets presented in this study can be found in online repositories. The names of the repository/repositories and accession number(s) can be found below: https://www.ncbi.nlm.nih.gov/, PRJNA764425.

## Author Contributions

CV, RK, and YB participated in the design of the study. CV performed the experiments and data analysis. Data interpretation was done by CV and RK. CV wrote the first draft and revised the manuscript based on the comments of RK. All authors contributed to the article and approved the submitted version.

## Funding

This research was financially supported by NWO-CNPq within the Joint Research Project Biobased Economy (729.004.011).

## Conflict of Interest

The authors declare that the research was conducted in the absence of any commercial or financial relationships that could be construed as a potential conflict of interest.

## Publisher's Note

All claims expressed in this article are solely those of the authors and do not necessarily represent those of their affiliated organizations, or those of the publisher, the editors and the reviewers. Any product that may be evaluated in this article, or claim that may be made by its manufacturer, is not guaranteed or endorsed by the publisher.
