# Supplementary material for: Small RNA Profiling of Susceptible and Resistant Ty-1 Encoding Tomato Plants Upon Tomato Yellow Leaf Curl Virus Infection
Source: Front Plant Sci. 2021 Nov 18;12:757165. doi: 10.3389/fpls.2021.757165 (PMC8637622; doi:10.3389/fpls.2021.757165)
Supplement: Supplementary file 1 [file Data_Sheet_1.docx]

**Supplementary Material**

**Supplementary Figures and Tables**

**Figure S1: Distribution of the 5’ terminal nucleotide of sense and antisense vsiRNA reads from MM and Ty-1 tomato.**
Viral siRNAs isolated from TYLCV infected MM or Ty-1 tomato, depicted per size class of 21-nt, 22-nt and 24-nt, and polarity, and showing the percentage of reads containing adenine (A), cytosine (C), guanine (G), or uracil (U) as 5’terminal nucleotide. The bar represents the average and standard deviation of three biological replicates.

**Figure S2: The distribution of vsiRNAs on the TYLCV genome in three biological replicates of MM and Ty-1 plants**Viral siRNAs isolated from infected MM (left panel) or Ty-1 bearing (right panel) tomato were mapped on the genome of tomato yellow leaf curl virus (TYLCV). The number of reads at each nucleotide position of the TYLCV genome is plotted for each sample (three biological replicates). Blue bars represent sense reads starting at each respective nucleotide, while the orange bars represent antisense reads ending at that position. The depicted percentage in the title represents the proportion that belongs to the size class in that specific sample relative to all vsiRNAs. A schematic overview of the TYLCV genome is depicted above the graphs, with the viral ORFs indicated as grey arrows and the intergenic region at the left and right side. The vsiRNA distribution profile is depicted for **(A)** the 21-nt size-class, **(B)** the 22-nt size-class, **(C)** the 24-nt size-class and **(D)** all vsiRNAs.

**Figure S3: Genomic distribution of vsiRNAs, depicted as percentage of the total vsiRNAs of that size class at each nucleotide position in MM and Ty-1 tomato.**
Viral siRNAs isolated from infected MM (left panel) or Ty-1 bearing (right panel) tomato are mapped on the genome of tomato yellow leaf curl virus (TYLCV). Per size class, the vsiRNAs are depicted as percentage of the total reads at each nucleotide position of the TYLCV genome (average from three biological replicates). Blue bars represent sense reads starting at each respective nucleotide, while the orange bars represent antisense reads ending at that position. The percentage in the title represents the proportion that belongs to the size class of all vsiRNAs in either MM or Ty-1 plants. A schematic overview of the TYLCV genome is presented at the top of each graph, with the viral ORFs indicated as grey arrows and the intergenic region at the left and right side.

**Figure S4: Percentage of vsiRNA reads per 100 nucleotides in the TYLCV genome.**
Viral siRNAs isolated from infected MM (dark blue line) or Ty-1 bearing (light blue line) tomato are mapped on the genome of tomato yellow leaf curl virus (TYLCV). The percentages of vsiRNA reads per 100 nucleotides of the TYLCV genome (amount of reads for position 1-100, 101-200 etc.) relative to the total amount of viral reads of the depicted size class are plotted. The graphs represent the average of three biological replicates. From each set of panels, the upper panel presents the sense and antisense polarities taken together, while the lower panel presents both polarities specified. A schematic overview of TYLCV genome is depicted at the top of each graph, with the viral ORFs indicated as grey arrows and the intergenic region at the left and right side. The genome distribution is depicted for **(A)** 21-nt, **(B)** 22-nt, **(C)** 24-nt and **(D)** all vsiRNAs.

**Figure S5**: **Potential viral sidRNAs generated during TYLCV-infection of MM and Ty-1 tomato**
Viral siRNAs isolated from TYLCV-infected MM or Ty-1 bearing tomato, were sequenced and subsequently mapped on the viral genome. **(A)** Graphical presentation of the amount of sense and antisense orientated viral reads within the size range of 25-33 nt from MM and Ty-1 tomato. The average of three biological replicates is depicted with the standard deviation. **(B)** Graph presenting the number of potential viral sidRNAs in MM and Ty-1 tomato (average of three biological replicates with standard deviation). Potential viral sidRNAs were identified from spots in the TYLCV genome that are targeted by vsiRNAs from different size classes (25-33 nt) and sharing the same 5’end. **(C)** Potential viral sidRNAs identified in MM tomato and mapping to the TYLCV genome at position 1324 nt. **(D)** Schematical diagram of the TYLCV genome and location of potential sidRNAs highligthed in red boxes (see also **Table S1**). **(E)** Close-up of the distribution of vsiRNAs (size range 25-33 nt) on the TYLCV genome region 1275-1335 nt in MM biological replicate sample 2. The amount of reads starting with the 5’ end of the vsiRNAs at each nucleotide position of the TYLCV genome is plotted, in either sense or antisense orientation. With a red box the potential sidRNAs (in size range 25-33 nt) are indicated. **(F)** Close-up of the distribution of vsiRNAs (size range 25-33 nt) on the TYLCV genome region 2050-2100 nt in Ty-1 biological replicate sample 2. The amount of reads starting with the 5’ end of the vsiRNA at each nucleotide position of the TYLCV genome is plotted, in either sense or antisense orientation. With a red box the potential sidRNAs (in size range 25-33 nt) are indicated.

| **Table S1: Identification of potential sidRNAs and their starting position on the TYLCV genome (1-2781)** |
| --- |

| MM TYLCV 1 | | MM TYLCV 2 | | MM TYLCV 3 | |
| --- | --- | --- | --- | --- | --- |
| (+) | (-) | (+) | (-) | (+) | (-) |
|  | 1262 | 575 | 1258 | 575 | 1230 |
|  | 1279 | 694 | 1262 |  | 1279 |
|  | 1288 | 883 | 1279 |  | 1287 |
|  | 1324 |  | 1280 |  | 1324 |
|  | 1329 |  | 1287 |  | 1329 |
|  | 1332 |  | 1288 |  | 1333 |
|  |  |  | 1324 |  | 1567 |
|  |  |  | 1329 |  | 1649 |
|  |  |  | 1530 |  | 1796 |
|  |  |  | 1558 |  | 2001 |
|  |  |  | 1796 |  | 2347 |
|  |  |  | 2162 |  |  |
| Ty-1 TYLCV 1 | | Ty-1 TYLCV 2 | | Ty-1 TYLCV 3 | |
| (+) | (-) | (+) | (-) | (+) | (-) |
| 627 | 2094 | 627 | 929 |  | 495 |
|  |  |  | 2056 |  | 930 |
|  |  |  | 2094 |  | 2094 |
|  |  |  | 2750 |  |  |
| Overview of potential sidRNA and their position in the TYLCV genome (nucleotide number 1-2781, sense or antisense orientation). Highlighted in yellow are the spots with potential sidRNAs that are found in all three biological replicates. | | | | | |
