# Supplementary figures and images for: Small RNA Profiling of Susceptible and Resistant Ty-1 Encoding Tomato Plants Upon Tomato Yellow Leaf Curl Virus Infection
Source: Front Plant Sci. 2021 Nov 18;12:757165. doi: 10.3389/fpls.2021.757165 (PMC8637622; doi:10.3389/fpls.2021.757165)

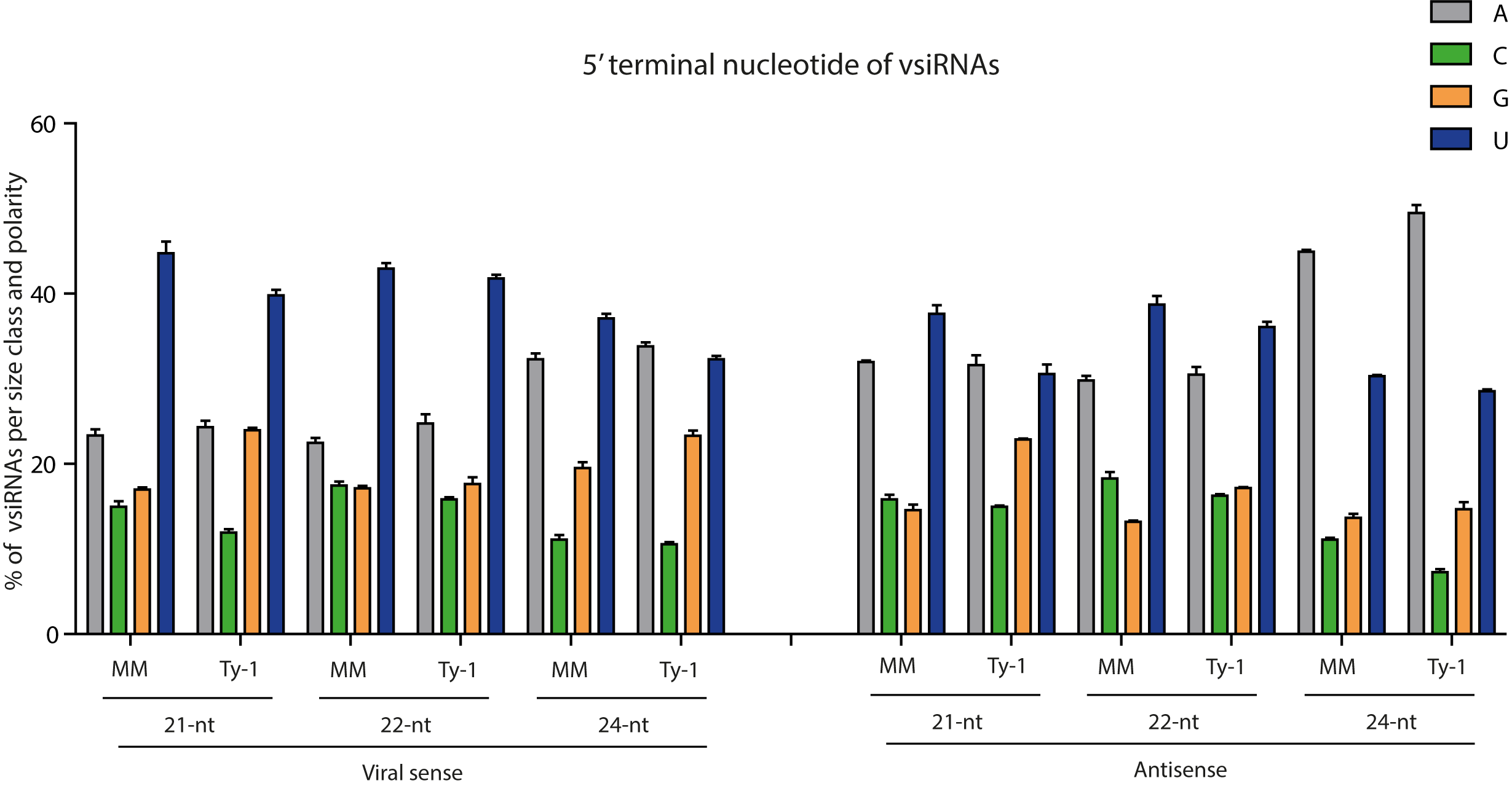

Supplement: Supplementary file 2 [file Image_1.TIF]

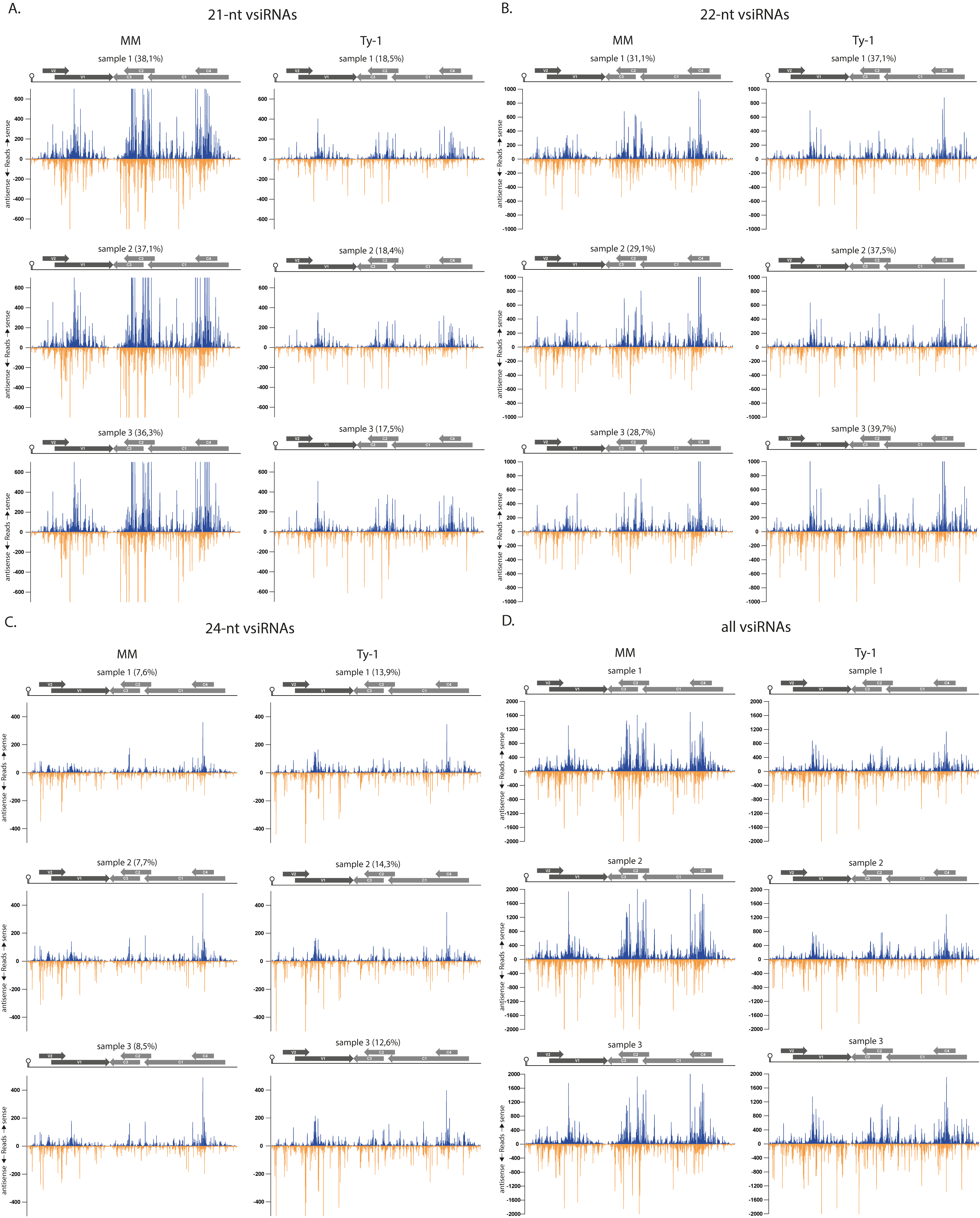

Supplement: Supplementary file 3 [file Image_2.TIF]

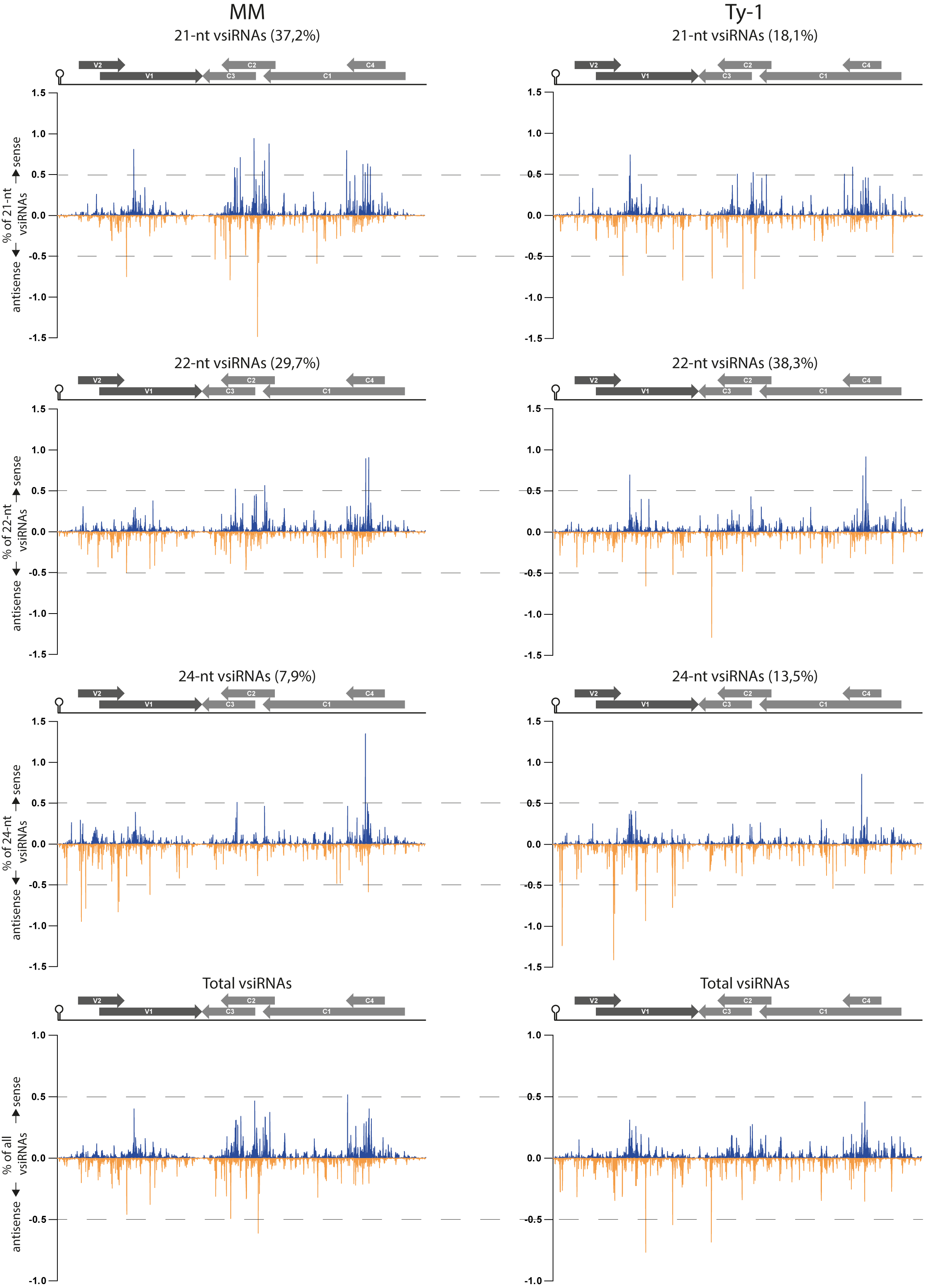

Supplement: Supplementary file 4 [file Image_3.TIF]

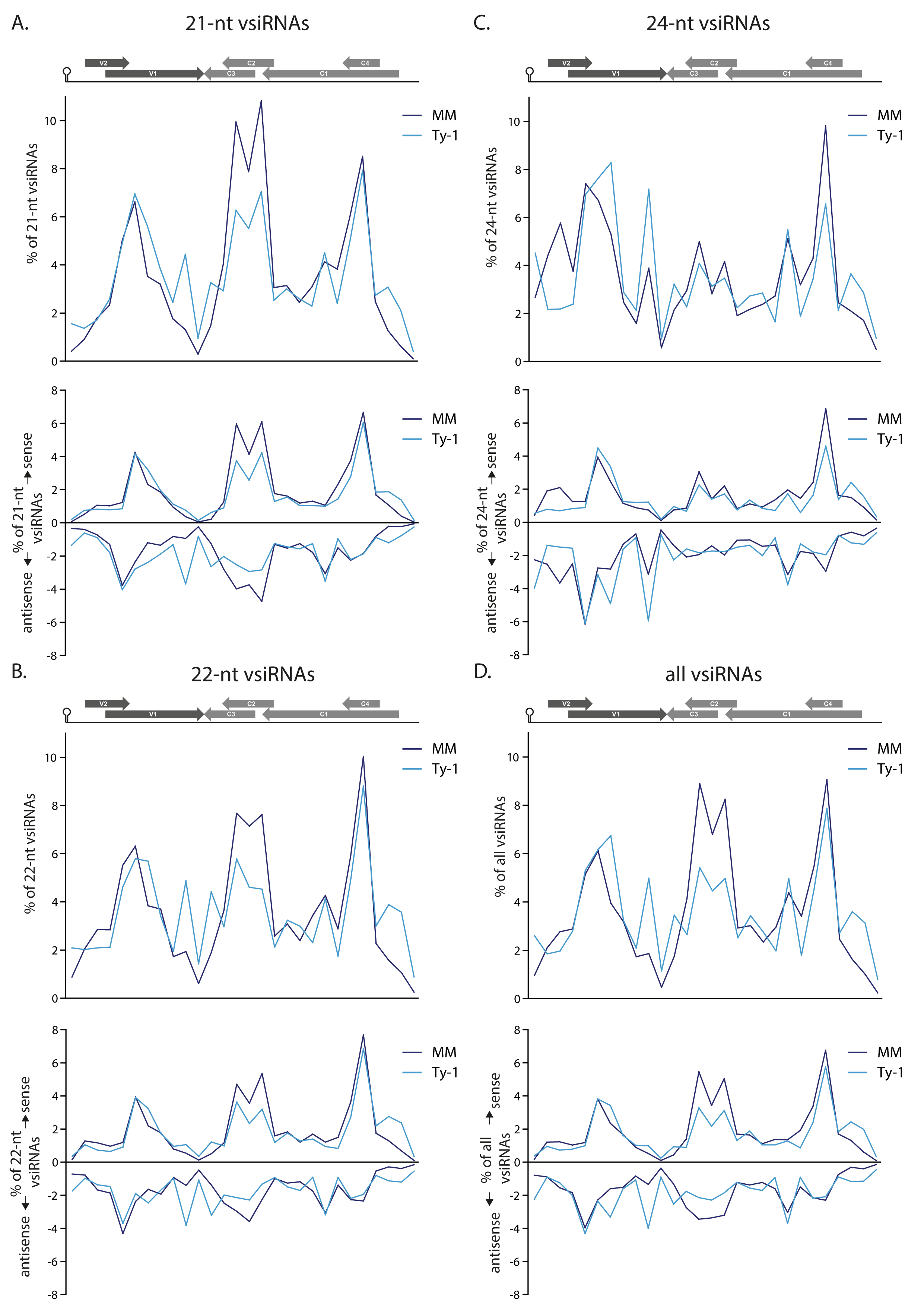

Supplement: Supplementary file 5 [file Image_4.TIF]

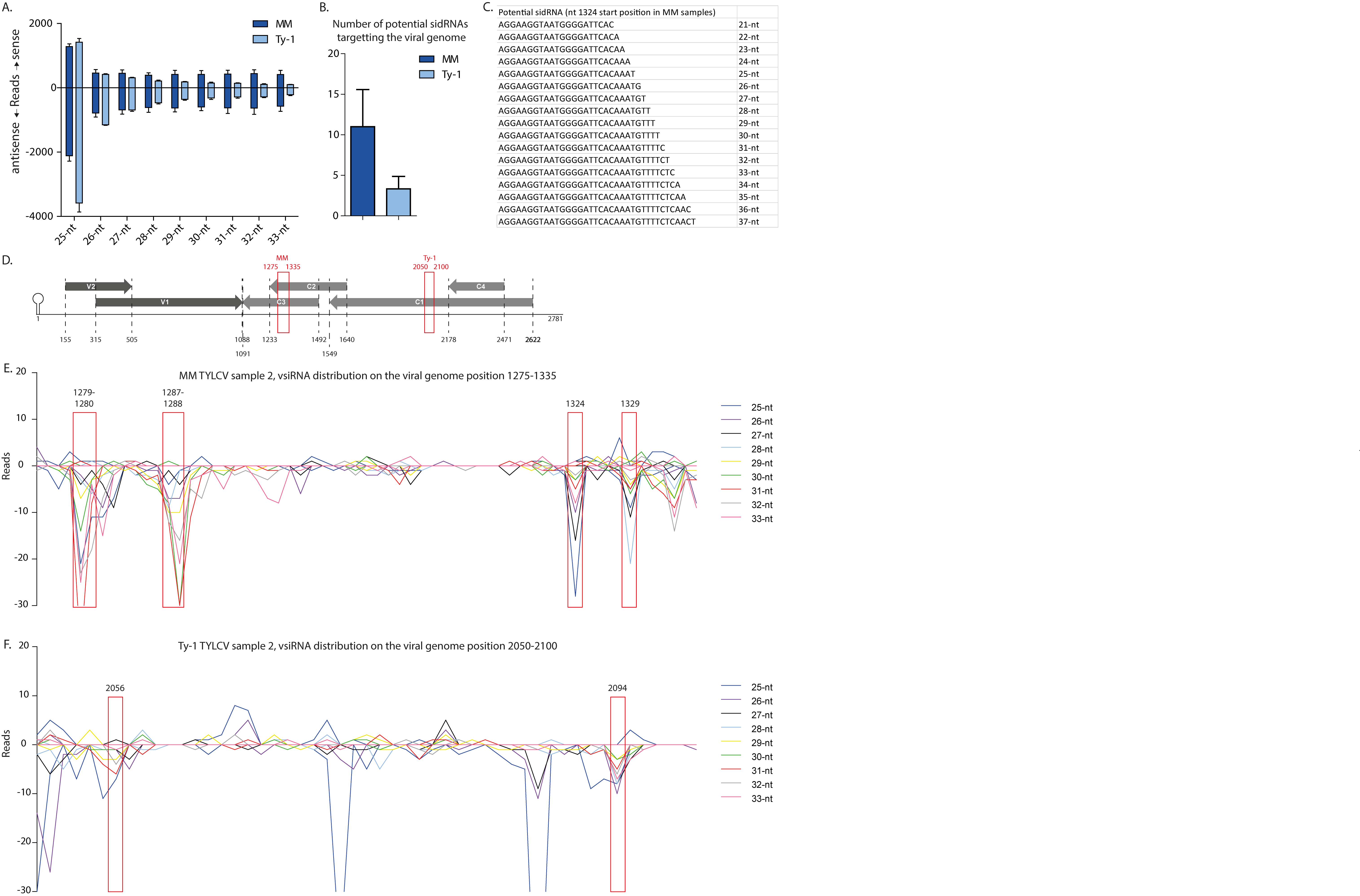

Supplement: Supplementary file 6 [file Image_5.TIF]
